# Supplementary material for: Transcription factor NFYA directs male meiotic entry by regulating accessible chromatin at meiotic promoters in mice
Source: EMBO J. 2026 Mar 19;45(8):2523–60. doi: 10.1038/s44318-026-00756-6 (PMC13083884; doi:10.1038/s44318-026-00756-6)
Supplement: Supplementary file 10 — Source data Fig. 2 [file 44318_2026_756_MOESM10_ESM.zip › Figure 2/2C/README.rtf]

Immunohistochemestry against NFYA. Images were adjusted for brightness with Photoshop, cropped and rotated in Adobe Illustrator. 
