# Supplementary figures and images for: Transcription factor NFYA directs male meiotic entry by regulating accessible chromatin at meiotic promoters in mice
Source: EMBO J. 2026 Mar 19;45(8):2523–60. doi: 10.1038/s44318-026-00756-6 (PMC13083884; doi:10.1038/s44318-026-00756-6)

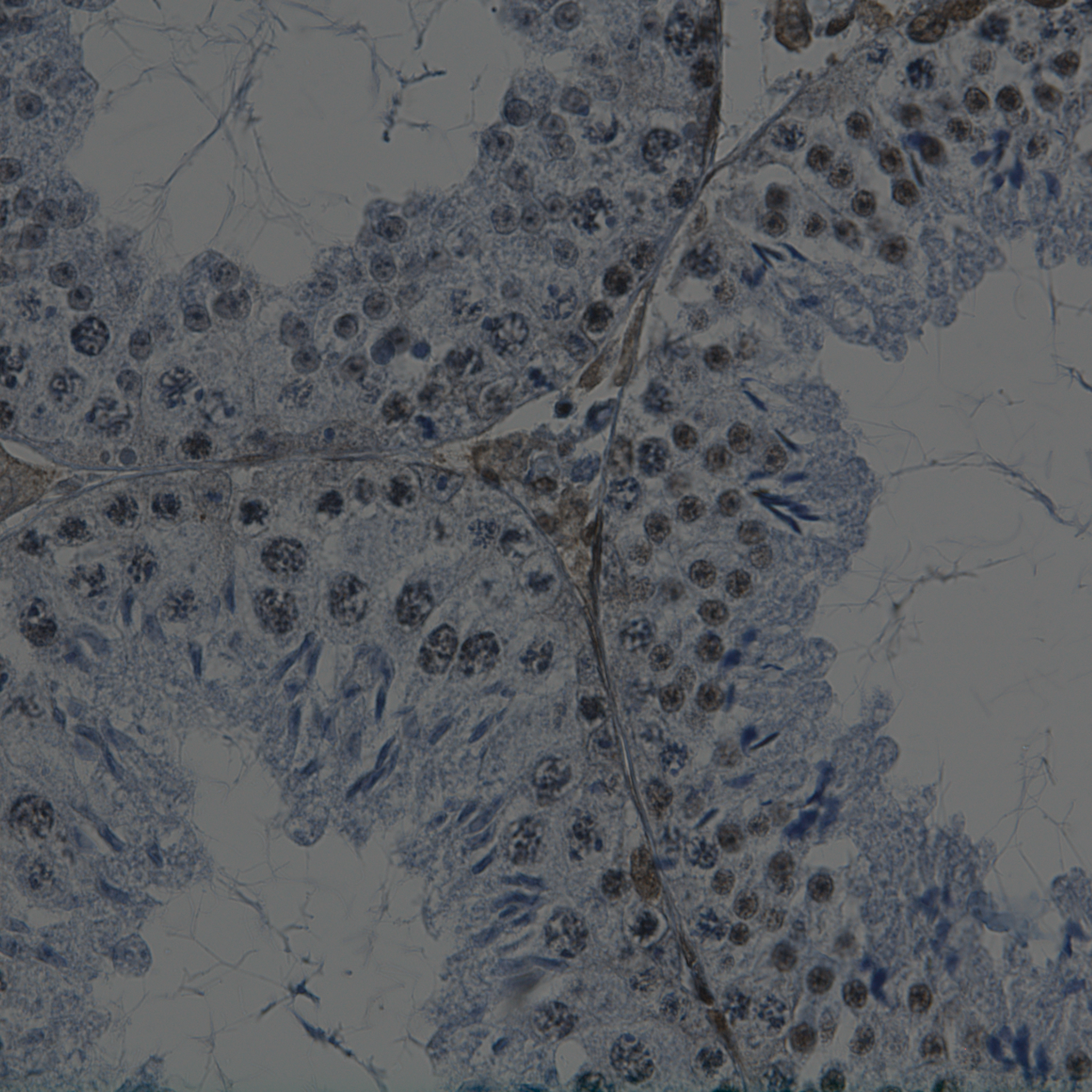

Supplement: Supplementary file 10 — Source data Fig. 2 [file 44318_2026_756_MOESM10_ESM.zip › Figure 2/2C/40x_WT-NFYA-2_stage8_9.tif]

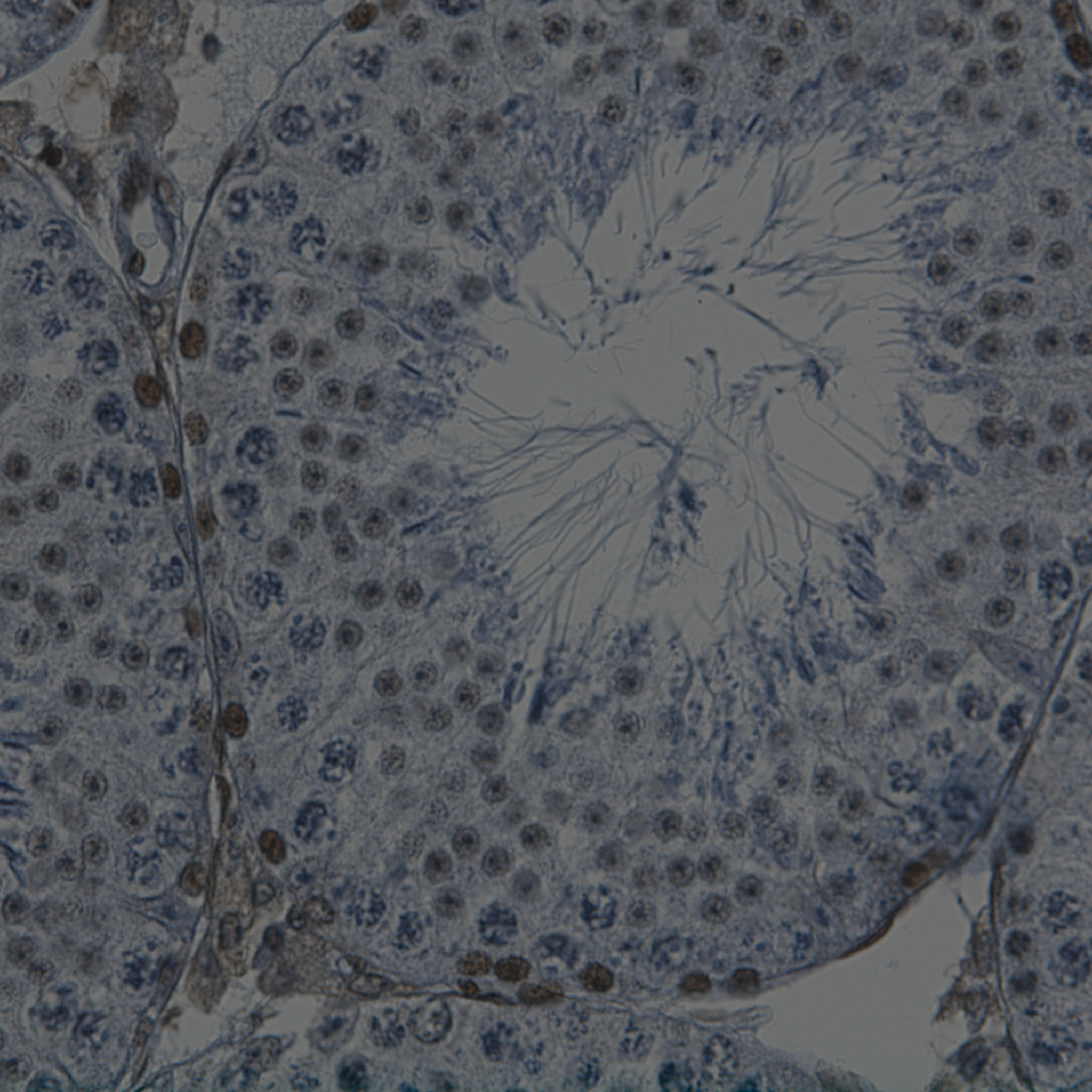

Supplement: Supplementary file 10 — Source data Fig. 2 [file 44318_2026_756_MOESM10_ESM.zip › Figure 2/2C/40x_WT-NFYA-13_stage3.tif]

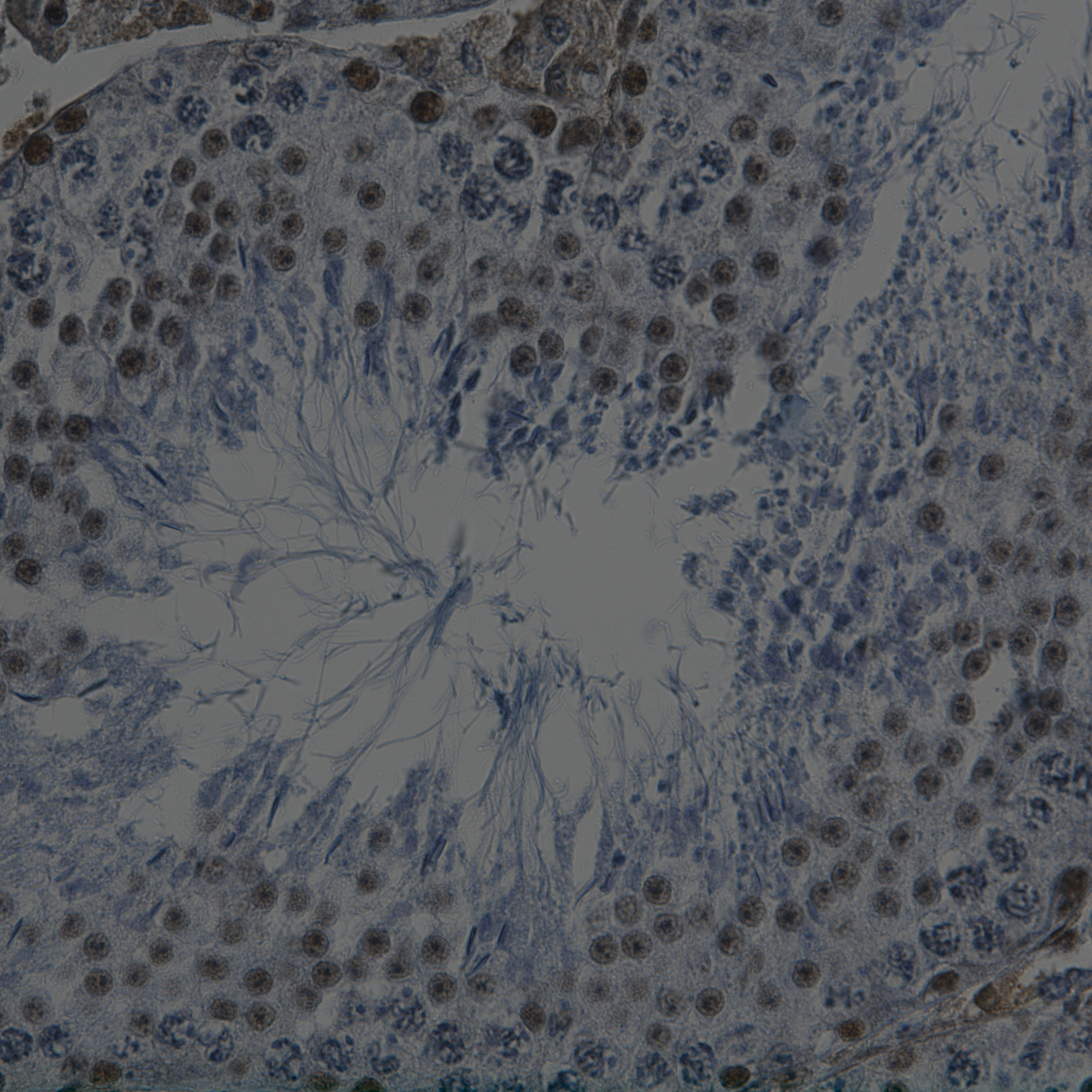

Supplement: Supplementary file 10 — Source data Fig. 2 [file 44318_2026_756_MOESM10_ESM.zip › Figure 2/2C/40x_WT-NFYA-1_stage6.tif]

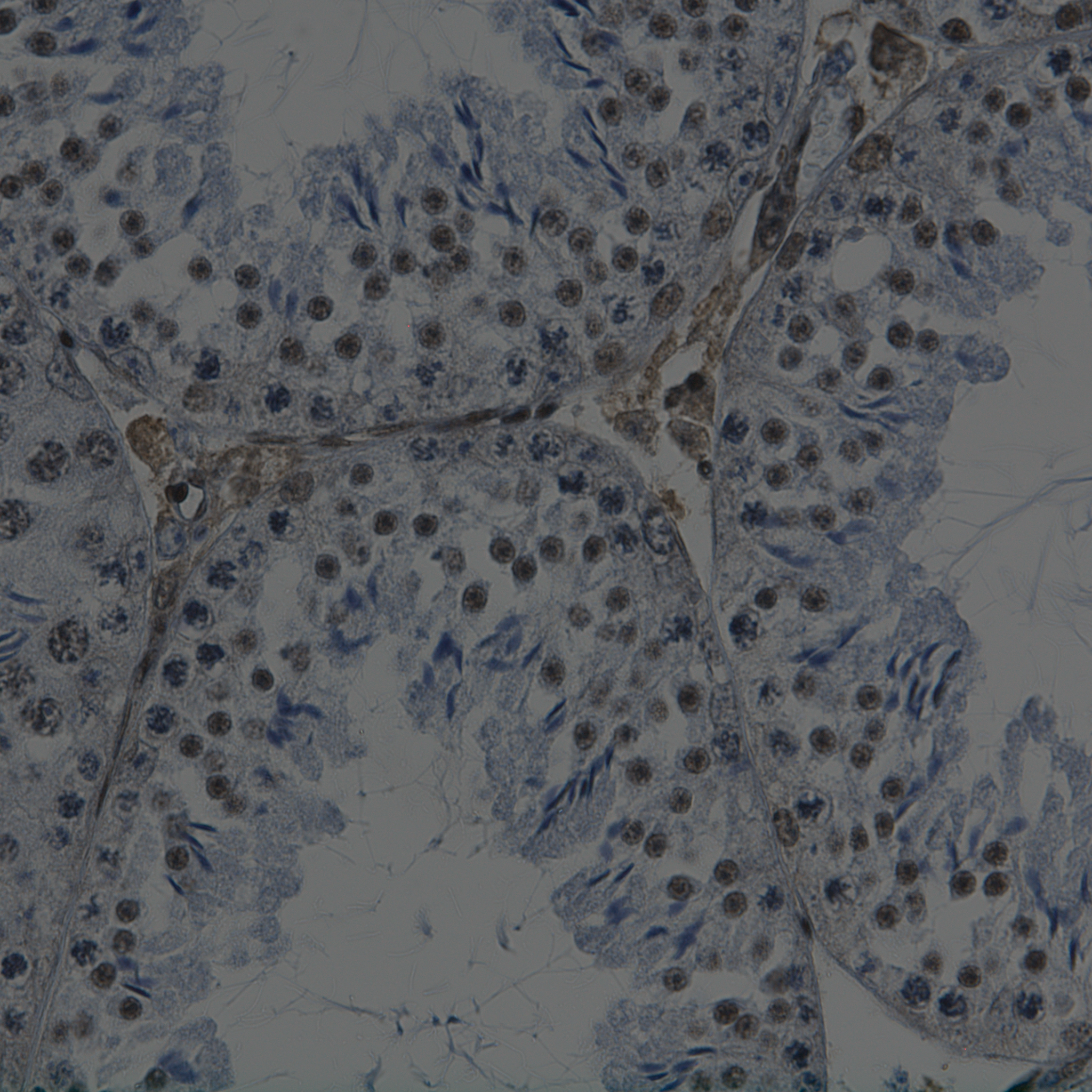

Supplement: Supplementary file 10 — Source data Fig. 2 [file 44318_2026_756_MOESM10_ESM.zip › Figure 2/2C/40x_WT-NFYA-9_stage2.tif]

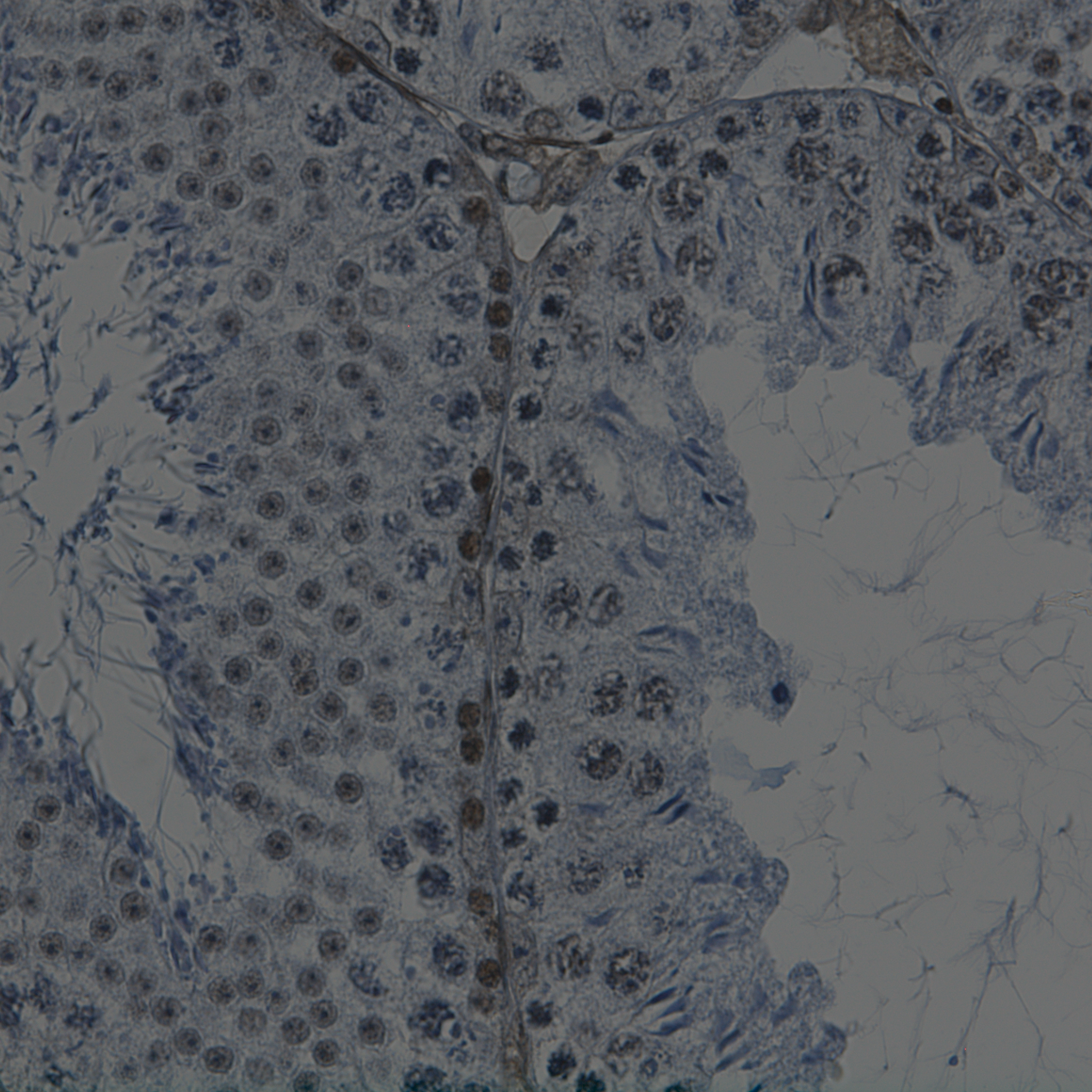

Supplement: Supplementary file 10 — Source data Fig. 2 [file 44318_2026_756_MOESM10_ESM.zip › Figure 2/2C/40x_WT-NFYA-6_stage11.tif]

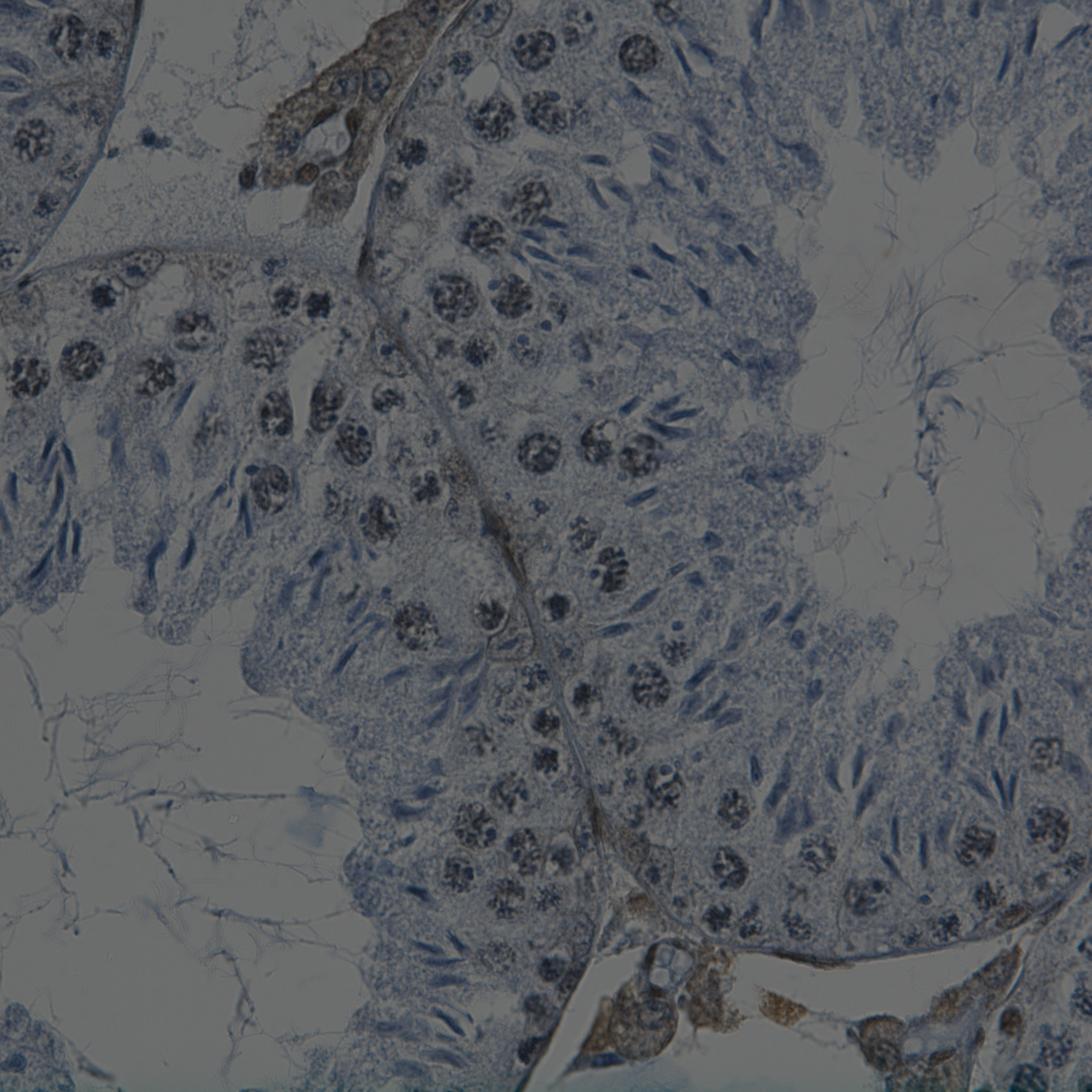

Supplement: Supplementary file 10 — Source data Fig. 2 [file 44318_2026_756_MOESM10_ESM.zip › Figure 2/2C/40x_WT-NFYA-3_stage10.tif]

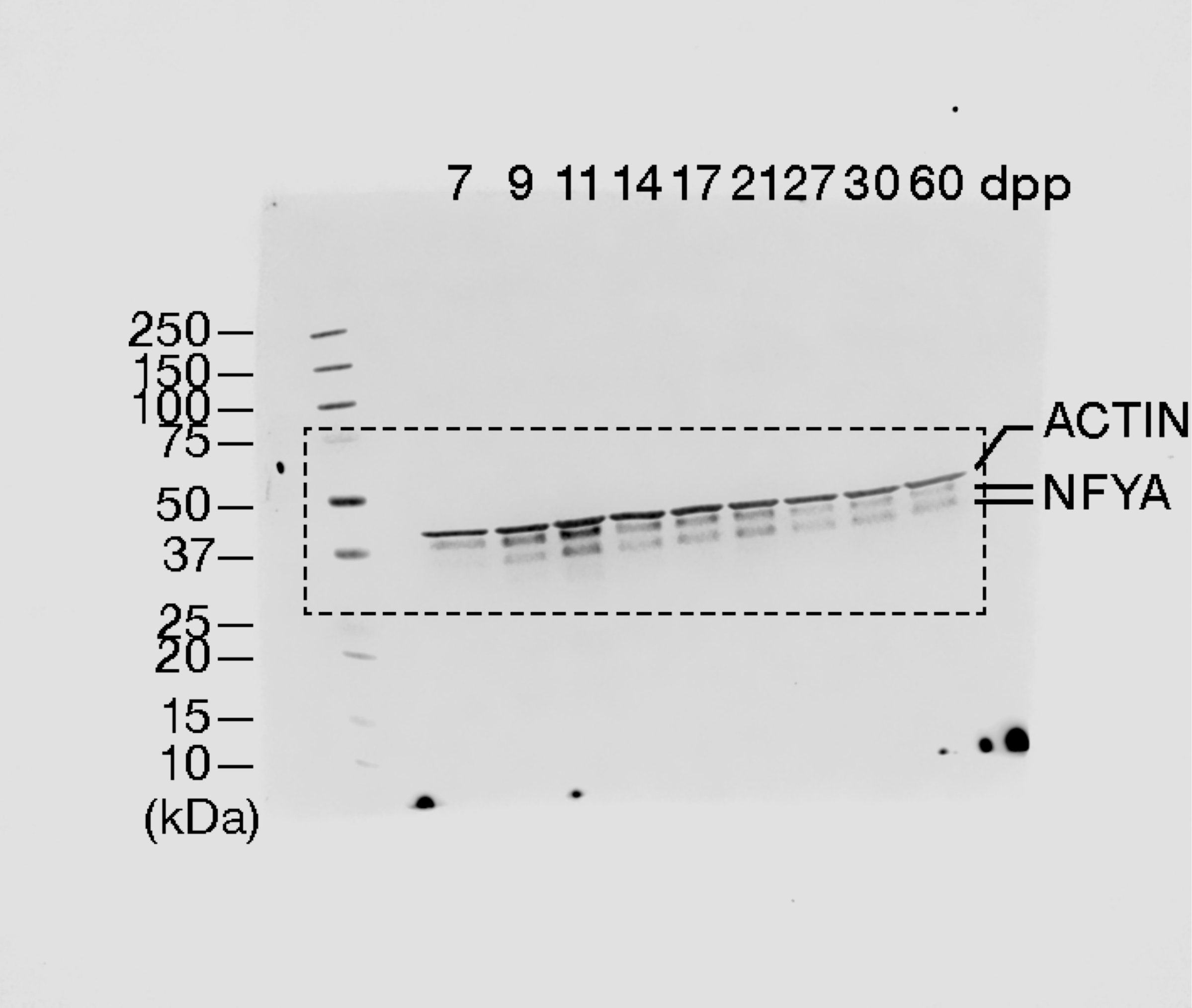

Supplement: Supplementary file 10 — Source data Fig. 2 [file 44318_2026_756_MOESM10_ESM.zip › Figure 2/2B/Westernblot replicate2.tif]

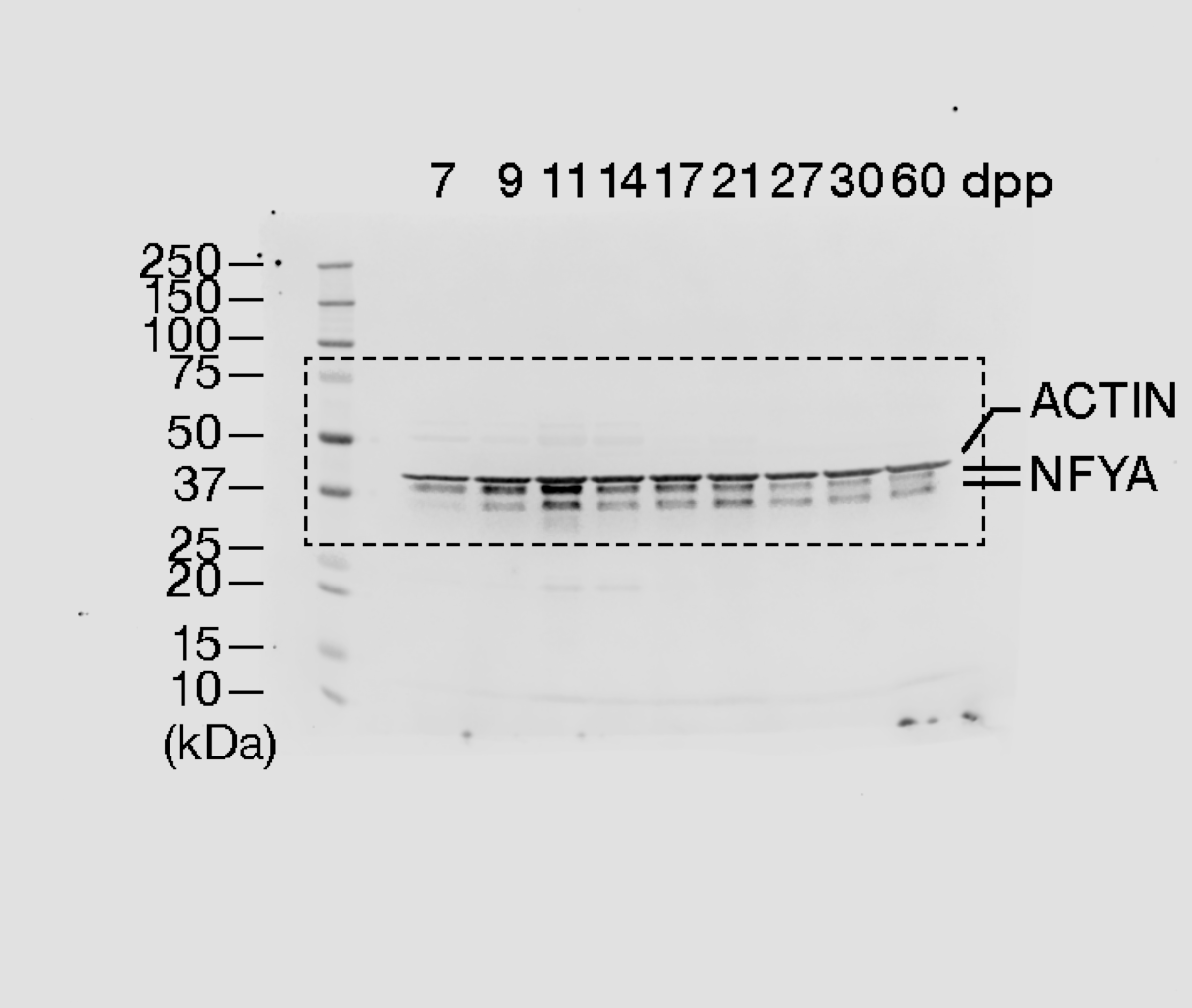

Supplement: Supplementary file 10 — Source data Fig. 2 [file 44318_2026_756_MOESM10_ESM.zip › Figure 2/2B/Westernblot replicate1.tif]
