# Supplementary material for: Transcription factor NFYA directs male meiotic entry by regulating accessible chromatin at meiotic promoters in mice
Source: EMBO J. 2026 Mar 19;45(8):2523–60. doi: 10.1038/s44318-026-00756-6 (PMC13083884; doi:10.1038/s44318-026-00756-6)
Supplement: Supplementary file 11 — Expanded View Figures [file 44318_2026_756_MOESM11_ESM.pdf]

Expanded View Figures

Figure EV1. Paused polymerase accumulates around the promoters of genes expressed during meiosis in spermatogonia.

Related to Fig. 1. (**A**) Violin plots represent the distance (bp) from TSS to nearest promoter PRO-seq signal of poised and non-poised meiosis-I (left) and spermiogenesis (right) genes in spermatogonia (SpG), primary spermatocytes (SpI), and round spermatids (RS). Horizontal lines represent the mean. Whiskers represent maximum and minimum values. Interquartile range (IQR) represented by boxplots. (**B**) Heatmaps show the relative promoter (left; from TSS to first 5% of gene length) and gene body (right; from first 30% of gene length to TES) PRO-seq signal for mitosis genes in spermatogonia (SpG), primary spermatocytes (SpI), and round spermatids (RS). (**C**) Line plots represent the average promoter (dashed line with triangle) and gene body (line with squares) relative PRO-seq signal for mitosis, meiosis-I, and spermiogenesis genes across SpG, SpI, and RS cells.

 © The Author(s)

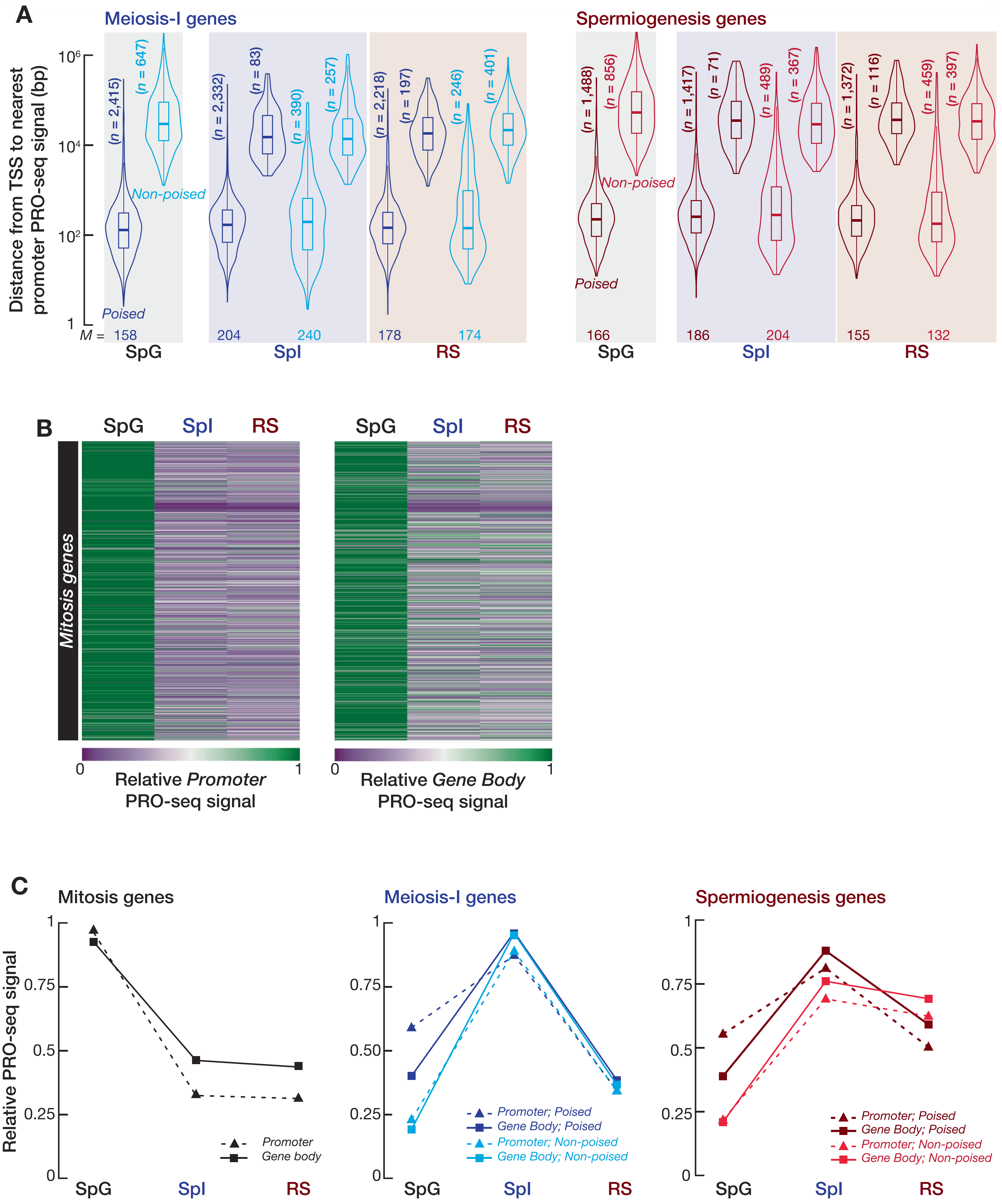
A
Meiosis-I genes
Spermiogenesis genes
Distance from TSS to nearest promoter PRO-seq signal (bp)
10^6
10^4
10^2
1
(n = 2,415)
(n = 647)
(n = 2,332)
(n = 83)
(n = 390)
(n = 257)
(n = 2,218)
(n = 197)
(n = 246)
(n = 401)
Non-poised
Poised
M = 158
204
240
178
174
SpG
SpI
RS
(n = 1,488)
(n = 856)
(n = 1,417)
(n = 71)
(n = 489)
(n = 367)
(n = 1,372)
(n = 116)
(n = 459)
(n = 397)
Non-poised
Poised
166
186
204
155
132
SpG
SpI
RS
B
SpG
SpI
RS
Mitosis genes
0
1
Relative Promoter PRO-seq signal
Relative Gene Body PRO-seq signal
C
Mitosis genes
Meiosis-I genes
Spermiogenesis genes
Relative PRO-seq signal
1
0.75
0.5
0.50
0.25
0
Promoter
Gene body
Promoter; Poised
Gene Body; Poised
Promoter; Non-poised
Gene Body; Non-poised
SpG
SpI
RS

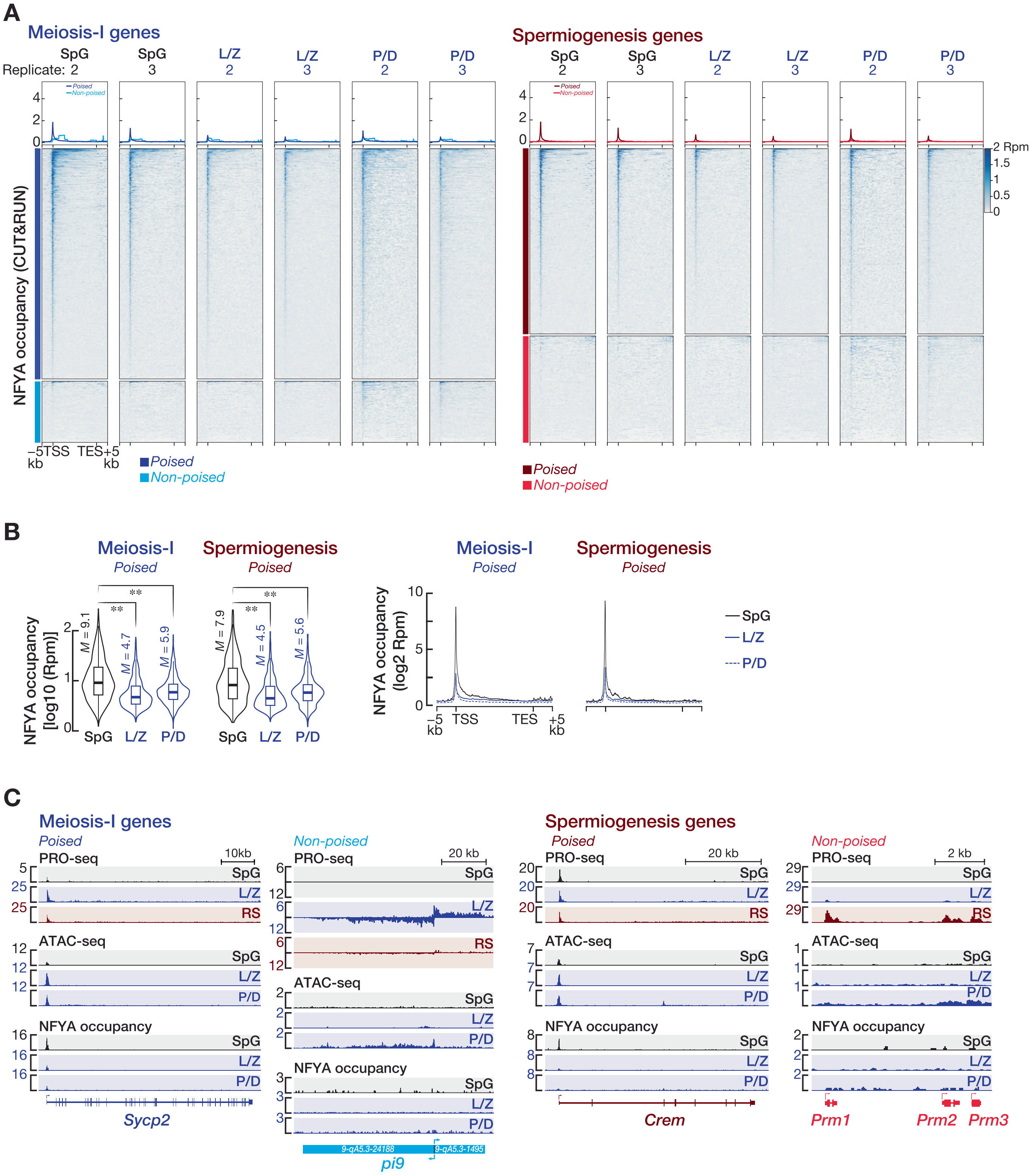
A
Meiosis-I genes
Spermiogenesis genes
Replicate: 2
SpG
L/Z
P/D
Poised
Non-poised
NFYA occupancy (CUT&RUN)
−5 kb TSS TES +5 kb
2 Rpm
B
Meiosis-I Poised
Spermiogenesis Poised
NFYA occupancy [log10 (Rpm)]
M = 9.1
M = 4.7
M = 5.9
M = 7.9
M = 4.5
M = 5.6
NFYA occupancy (log2 Rpm)
SpG
L/Z
P/D
C
Meiosis-I genes
Spermiogenesis genes
Poised
Non-poised
PRO-seq
ATAC-seq
NFYA occupancy
RS
10kb
20 kb
2 kb
Sycp2
pi9
Crem
Prm1
Prm2
Prm3

 © The Author(s)

Figure EV2. NFYA binds the promoters of poised genes in spermatogonia.

Related to Fig. 2. (**A**) Metagene plots (top) and heatmaps (bottom) show Rpm-normalized NFYA CUT&RUN signals in the −5 kb to +5 kb window flanking transcription start sites (TSSs) and transcription end sites (TESs) of poised and non-poised genes in the second and third replicates from SpG, L/Z, P/D. (**B**) Violin plots (left) show NFYA occupancy around the promoters of poised meiosis-I and spermiogenesis genes in SpG, L/Z, and P/D. Horizontal lines represent the median. Whiskers represent maximum and minimum values. IQR represented by boxplots. $^{**}P < 0.01$; two-sided Wilcoxon matched-pairs signed-ranked sum test. Meiosis-I genes in SpG vs L/Z $P < 2.2 \times 10^{-16}$, SpG vs P/D $P = 1.566 \times 10^{-249}$; Spermiogenesis genes in SpG vs L/Z $P = 1.474 \times 10^{-220}$, SpG vs P/D $P = 3.384 \times 10^{-131}$. Metagene plots (right) show Rpm-normalized NFYA CUT&RUN signals in the −5 kb to +5 kb window flanking transcription start sites (TSSs) and transcription end sites (TESs) of poised and non-poised genes in the second and third replicates from SpG, L/Z, P/D. Average of three biological replicates ($n = 3$). (**C**) IGV tracks of PRO-seq signal, ATAC-seq signal, and NFYA occupancy (CUT&RUN) at gene boundaries of exemplified poised and non-poised meiosis-I (*Sycp2, pi9*) and spermiogenesis (*Crem, Prm1, Prm2, Prm3*) genes from SpG, L/Z, P/D, and RS.

© The Author(s)

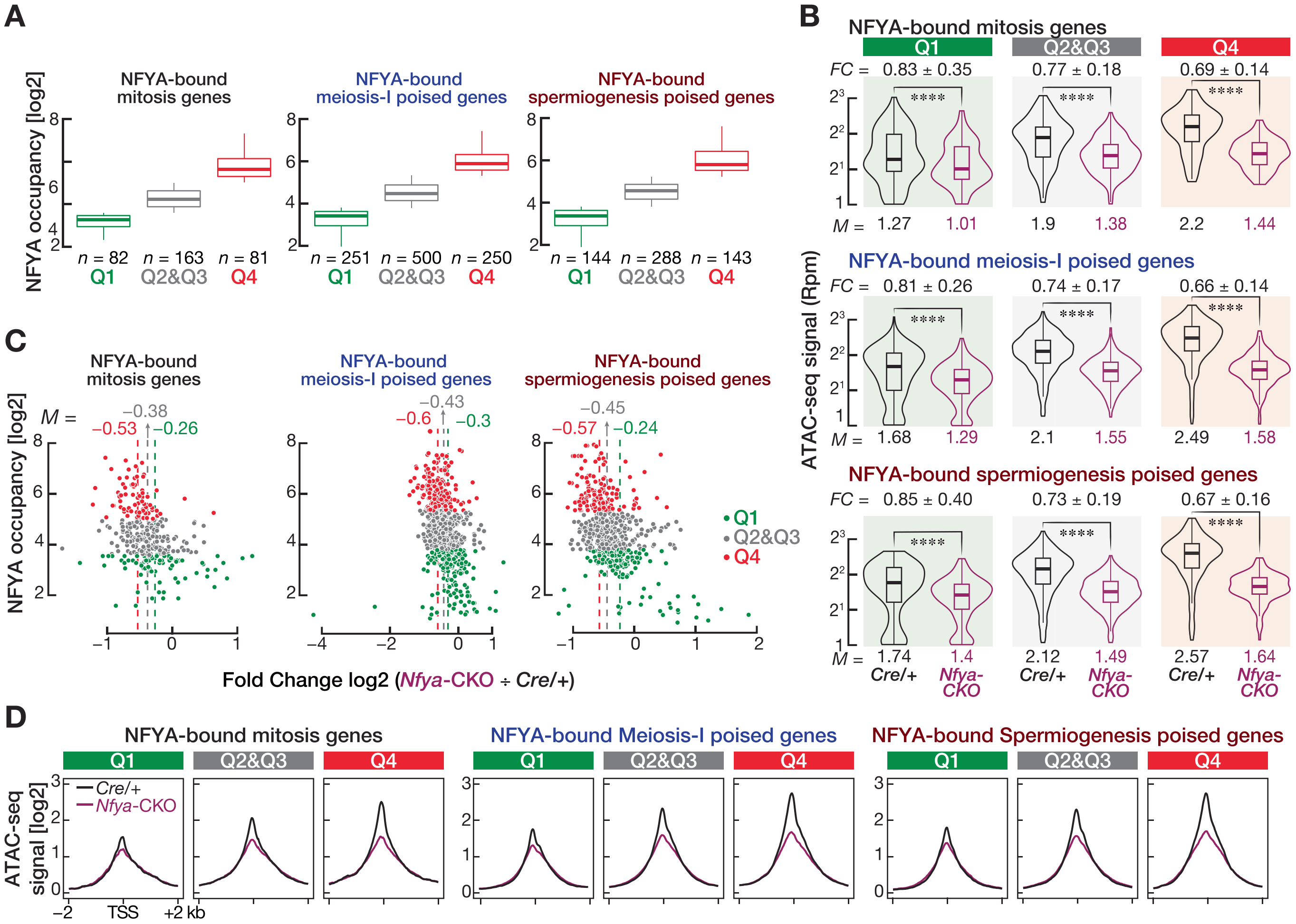

Figure EV3. Degree of NFYA occupancy correlates with the degree of reduction in accessible chromatin.

Related to Fig. 5. (**A**) Boxplots show rank-ordered NFYA-bound mitosis, meiosis-I and spermiogenesis poised genes relative to the NFYA occupancy at their promoters in SpG of *Cre/+* mice. Q1 denotes the first quartile; Q2 and Q3 represent the second and third quartiles; Q4 represents the fourth quartile. Horizontal lines represent the mean. Whiskers represent maximum and minimum values. IQR represented by boxplots. (**B**) Reads per million (Rpm)-normalized ATAC-seq signals at the promoters of NFYA-bound mitosis, poised meiosis-I, and poised spermiogenesis genes that are classified according to quartiles (NFYA-bound mitosis genes, Q1, $n = 82$, Q2&Q3, $n = 163$, Q4, $n = 81$; NFYA-bound meiosis-I genes, Q1, $n = 251$, Q2&Q3, $n = 500$, Q4, $n = 250$; NFYA-bound spermiogenesis genes, Q1, $n = 144$, Q2&Q3, $n = 288$, Q4, $n = 143$). FC fold change. Horizontal lines represent the mean. Whiskers represent maximum and minimum values. Interquartile range (IQR) represented by boxplots. Two-sided Wilcoxon matched-pairs signed-ranked sum test. ****$P < 0.0001$. NFYA-bound mitosis genes Q1 *Nfya-CKO* vs *Cre/+* $P = 7.032 \times 10^{-8}$, Q2&Q3 $P = 8.177 \times 10^{-25}$, Q4 $P = 9.549 \times 10^{-15}$; NFYA-bound meiosis-I poised genes Q1 *Nfya-CKO* vs *Cre/+* $P = 1.358 \times 10^{-29}$, Q2&Q3 $P = 4.496 \times 10^{-79}$, Q4 $P = 2.843 \times 10^{-42}$; NFYA-bound spermiogenesis poised genes Q1 *Nfya-CKO* vs *Cre/+* $P = 1.729 \times 10^{-17}$, Q2&Q3 $P = 2.152 \times 10^{-45}$, Q4 $P = 1.283 \times 10^{-24}$. (**C**) Scatter plots show the change in accessible chromatin between the SpG from *Nfya-CKO* and *Cre/+* relative to their NFYA occupancy. NFYA-bound genes are presented in quartiles. (**D**) Metagene plots of ATAC-seq signal (log2) around the TSS ± 2 kb of NFYA-bound mitosis, meiosis-I, and spermiogenesis poised genes in SpG of *Cre/+* and *Nfya-CKO* mice.

 © The Author(s)

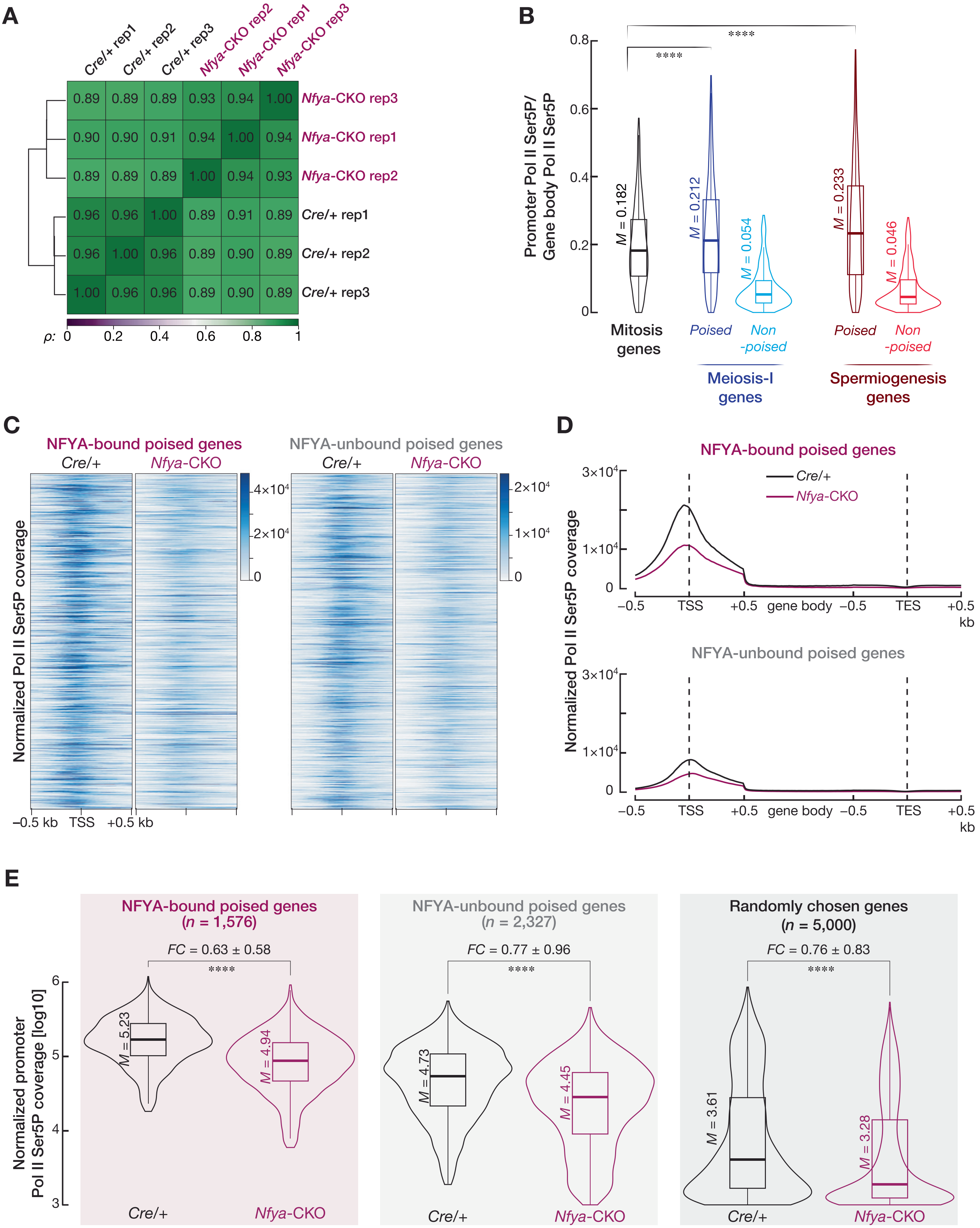
A
Cre/+ rep1
Cre/+ rep2
Cre/+ rep3
Nfya-CKO rep2
Nfya-CKO rep1
Nfya-CKO rep3
Nfya-CKO rep3
Nfya-CKO rep1
Nfya-CKO rep2
Cre/+ rep1
Cre/+ rep2
Cre/+ rep3
ρ: 0 0.2 0.4 0.6 0.8 1
B
Promoter Pol II Ser5P/ Gene body Pol II Ser5P
M = 0.182
M = 0.212
M = 0.054
M = 0.233
M = 0.046
Mitosis genes
Poised
Non -poised
Meiosis-I genes
Spermiogenesis genes
C
NFYA-bound poised genes
NFYA-unbound poised genes
Cre/+
Nfya-CKO
Normalized Pol II Ser5P coverage
–0.5 kb TSS +0.5 kb
D
NFYA-bound poised genes
NFYA-unbound poised genes
Normalized Pol II Ser5P coverage
–0.5 TSS +0.5 gene body –0.5 TES +0.5 kb
E
NFYA-bound poised genes (n = 1,576)
FC = 0.63 ± 0.58
M = 5.23
M = 4.94
NFYA-unbound poised genes (n = 2,327)
FC = 0.77 ± 0.96
M = 4.73
M = 4.45
Randomly chosen genes (n = 5,000)
FC = 0.76 ± 0.83
M = 3.61
M = 3.28
Normalized promoter Pol II Ser5P coverage [log10]
Cre/+
Nfya-CKO

Figure EV4. *Nfya* deletion impairs paused Pol II occupancy around the TSSs of poised genes in spermatogonia.

Related to Fig. 5. (**A**) Heatmap shows Spearman's correlation between the biological replicates of Pol II Ser5P CUT&Tag from SpG of *Cre/+* and *Nfya-CKO*. Agreement between replicates; SpG from *Cre/+* Spearman's $\rho > 0.95$; SpG from *Nfya-CKO* Spearman's $\rho > 0.92$. (**B**) Violin plots represent the density (average of three biological replicates) of Pol II Ser5P between +31 to +60 nt from the TSS relative to the Pol II Ser5P density at the gene body for poised and non-poised meiosis-I and spermiogenesis genes, and mitosis genes in SpG from *Cre/+* ($n = 3$). Horizontal lines represent the mean. Whiskers represent maximum and minimum values. Interquartile range (IQR) represented by boxplots. Two-sided Wilcoxon matched-pairs signed-rank sum test. ****$P < 0.0001$. Mitosis vs poised meiosis-I genes $P = 1.253 \times 10^{-13}$, mitosis vs poised spermiogenesis genes $P = 5.454 \times 10^{-9}$. (**C**) Heatmaps (average of three biological replicates) show the normalized Pol II Ser5P coverage around the TSSs of NFYA-bound and -unbound poised genes in SpG of *Cre/+* and *Nfya-CKO*. (**D**) Metagene plots (average of three biological replicates) show the normalized Pol II Ser5P coverage around the TSSs, gene body, and TESs of NFYA-bound and -unbound poised genes in SpG of *Cre/+* and *Nfya-CKO*. (**E**) Violin plots show the change in the *E. coli* spike-in normalized coverage of Pol II Ser5P within ±0.5 kb of the TSSs of NFYA-bound and -unbound poised genes and 5,000 randomly chosen genes in SpG of *Cre/+* and *Nfya-CKO*. Horizontal lines represent the mean. Whiskers represent maximum and minimum values. Interquartile range (IQR) represented by boxplots. Two-sided Wilcoxon matched-pairs signed-rank sum test. ****$P < 0.0001$. NFYA-bound poised genes *Cre/+* vs *Nfya-CKO* $P = 3.33 \times 10^{-98}$; NFYA-unbound poised genes *Cre/+* vs *Nfya-CKO* $P = 3.28 \times 10^{-70}$; Randomly chosen genes *Cre/+* vs *Nfya-CKO* $P = 1.77 \times 10^{-88}$.

 © The Author(s)

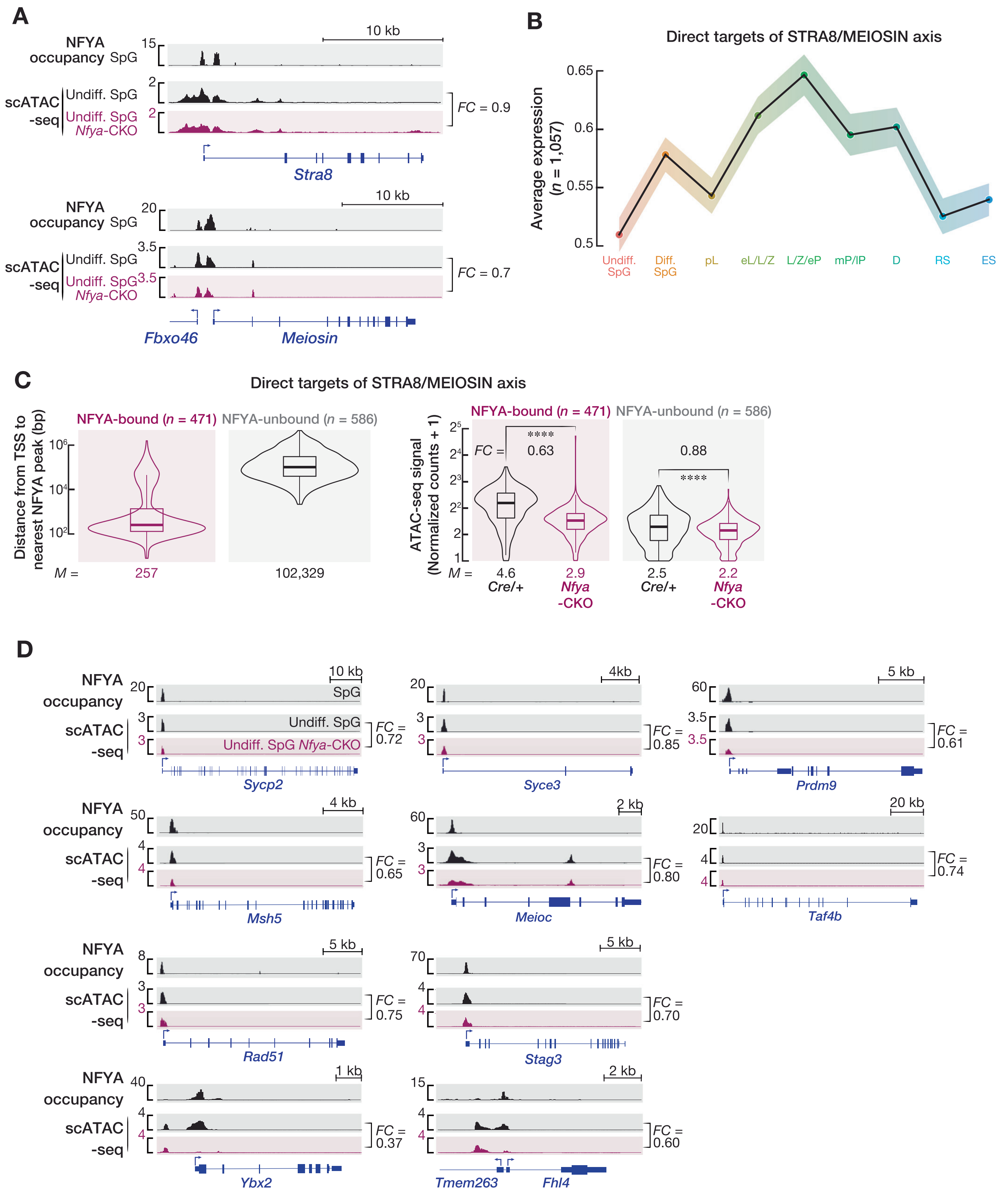
A
NFYA occupancy SpG
scATAC -seq
Undiff. SpG
Undiff. SpG Nfya-CKO
10 kb
FC = 0.9
Stra8
FC = 0.7
Fbxo46
Meiosin
B
Direct targets of STRA8/MEIOSIN axis
Average expression (n = 1,057)
0.65
0.6
0.55
0.5
Undiff. SpG
Diff. SpG
pL
eL/L/Z
L/Z/eP
mP/lP
D
RS
ES
C
Direct targets of STRA8/MEIOSIN axis
NFYA-bound (n = 471)
NFYA-unbound (n = 586)
Distance from TSS to nearest NFYA peak (bp)
M = 257
102,329
ATAC-seq signal (Normalized counts + 1)
FC = 0.63
0.88
****
4.6 Cre/+
2.9 Nfya-CKO
2.5 Cre/+
2.2 Nfya-CKO
D
NFYA occupancy
scATAC -seq
SpG
Undiff. SpG
Undiff. SpG Nfya-CKO
Sycp2 FC = 0.72
Syce3 FC = 0.85
Prdm9 FC = 0.61
Msh5 FC = 0.65
Meioc FC = 0.80
Taf4b FC = 0.74
Rad51 FC = 0.75
Stag3 FC = 0.70
Ybx2 FC = 0.37
Tmem263
Fhl4 FC = 0.60

Figure EV5. NFYA regulates accessible chromatin at the promoters of genes activated by the STRA8/MEIOSIN axis.

Related to Figs. 4, 5, and 6. (**A**). IGV tracks of NFYA occupancy (CUT&RUN) and scATAC-seq signal, at gene boundaries of *Stra8* and *Meiosisn* in SpG of *Cre/+* and *Nfya-CKO* mice. (**B**) Line plot of average expression of STRA8/MEIOSIN targets in nine different germ cell populations defined by scRNA-seq. (**C**) Violon plots of distance from TSS of nearest NFYA peak (left) of genes directly targeted of STRA8/MEIOSIN axis in SpG. ATAC-seq signal (right) of genes directly targeted of STRA8/MEIOSIN axis in SpG of *Cre/+* and *Nfya-CKO* mice. Horizontal lines represent the median. Whiskers represent maximum and minimum values. IQR represented by boxplots. ****$P < 0.0001$; two-sided Wilcoxon matched-pairs signed-ranked sum test. NFYA-bound *Cre/+* vs *Nfya-CKO* $P = 2.85 \times 10^{-70}$; NFYA-unbound *Cre/+* vs *Nfya-CKO* $P = 8.85 \times 10^{-32}$. (**D**) IGV tracks of NFYA occupancy (CUT&RUN) and scATAC-seq signal, at gene boundaries of genes that are direct targets of the STRA8/MEIOSIN axis.

 © The Author(s)
