# Supplementary material for: Transcription factor NFYA directs male meiotic entry by regulating accessible chromatin at meiotic promoters in mice
Source: EMBO J. 2026 Mar 19;45(8):2523–60. doi: 10.1038/s44318-026-00756-6 (PMC13083884; doi:10.1038/s44318-026-00756-6)
Supplement: Supplementary file 1 — Appendix [file 44318_2026_756_MOESM1_ESM.pdf]

Appendix

Table of content

Appendix Figure S1. Each major step of spermatogenesis retains a distinct gene expression profile. Related to Figure 1. 2

Appendix Figure S2. Strategy for purifying male germ cells using fluorescence-activated cell sorter (FACS). Related to Figures 1 and 2. 3

Appendix Figure S3. Poised genes have accessible chromatin around their promoter regions in spermatogonia. Related to Figures 1 and 2. 4

Appendix Figure S4. HOMER motif analysis around the promoters of poised genes. Related to Figure 2. 5

Appendix Figure S5. Temporal expression of NFYA in the testes of wild-type mouse. Related to Figure 2. 6

Appendix Figure S6. Correlation between NFYA CUT&RUN replicates. Related to Figure 2. 7

Appendix Figure S7. NFYA binds the promoters of poised genes in spermatogonia. Related to Figure 2. 8

Appendix Figure S8. Male germ cell-specific deletion of *Nfya*. Related to Figure 3. 9

Appendix Figure S9. NFYA deficient male germ cells have reduced testis size and no sperm in epididymis. Related to Figure 3. 10

Appendix Figure S10. NFYA deficient male germ cells arrest at meiotic entry. Related to Figure 3. 11

Appendix figure S11. Identification of cell populations of the testes from 8-week-old Cre/+ and Nfya-CKO mice along with wild-type staged mice using simultaneous single-cell (sc) RNA-seq and ATAC-seq. Related to Figures 4, 5, and 6. 12

Säflund et al., Appendix Figure S1

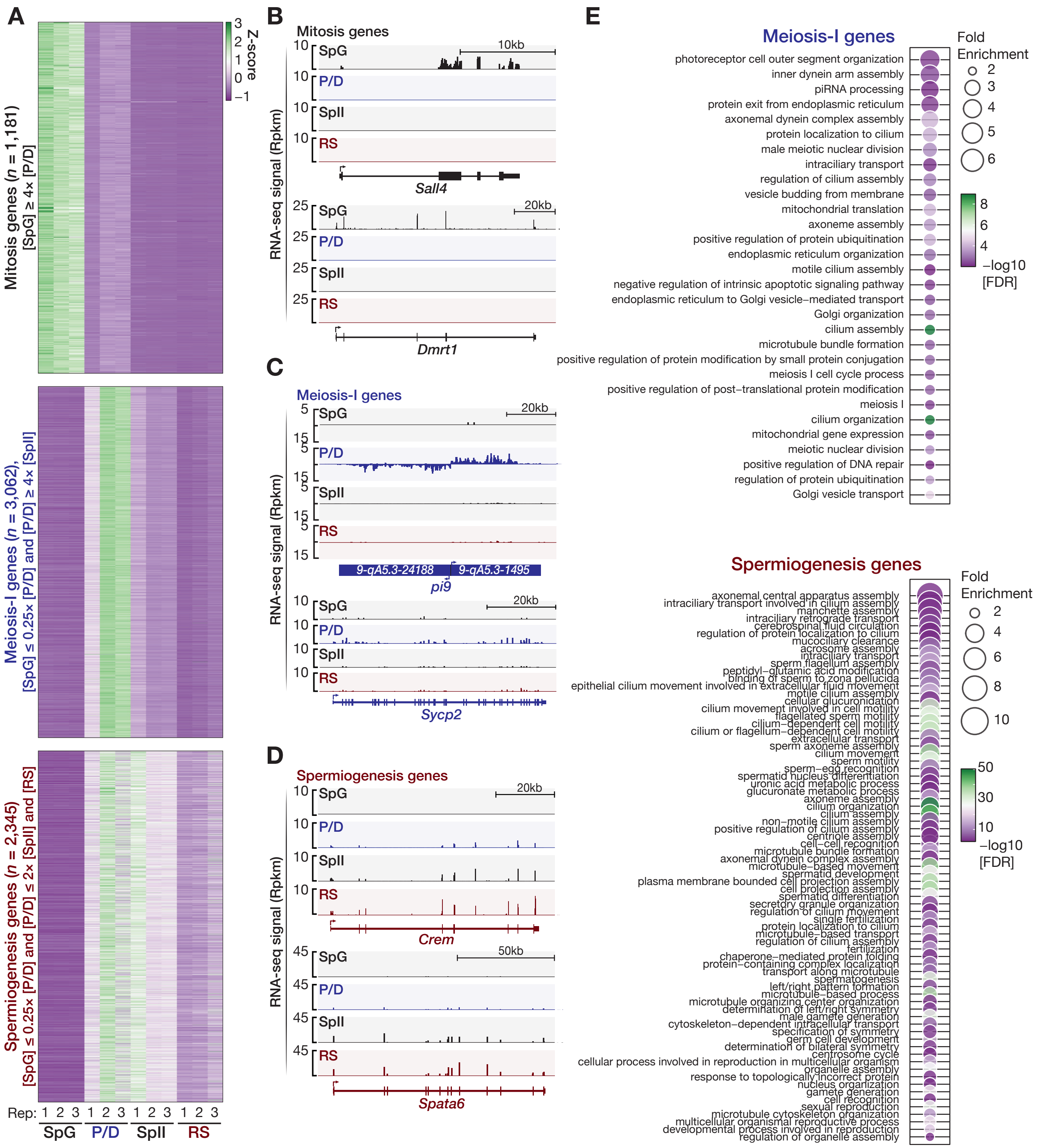

Appendix Figure S1. Each major step of spermatogenesis retains a distinct gene expression profile. Related to Figure 1.

(A) Heatmaps show absolute steady-state transcript abundance of mitosis, meiosis-I and spermiogenesis genes in spermatogonia (SpG), pachytene and diplotene (P/D), secondary spermatocytes (SpII) and round spermatids (RS). Genes whose RNA transcript abundance is ≥4-fold higher in SpG compared to P/D, SpII and RS are classified as mitosis specific genes. Meiosis-I genes are classified as those whose RNA transcript abundance is ≤0.25-fold lower in SpG compared to P/D and are ≥4-fold higher in P/D compared to SpII. Spermiogenesis genes are defined as those with ≤2-fold increases in SpII and RS compared to P/D and ≤0.25-fold lower in SpG compared to P/D.

(B- D) Integrated Genomics Viewer (IGV) views of reads per kilobase million (Rpkm)-normalized RNA sequencing signal for gene examples from **(B)** Mitosis genes, *Sall4, Dmrt1*, **(C)** Meiosis-I genes, *pi9*, *Sycp2*, **(D)** spermiogenesis genes, *Crem*, *Spata6*.

(E) Gene Ontology (GO) analysis of meiosis-I (top) and spermiogenesis (bottom) genes. The gene categories are sorted by fold enrichment.

Säflund et al., Appendix Figure S2

A

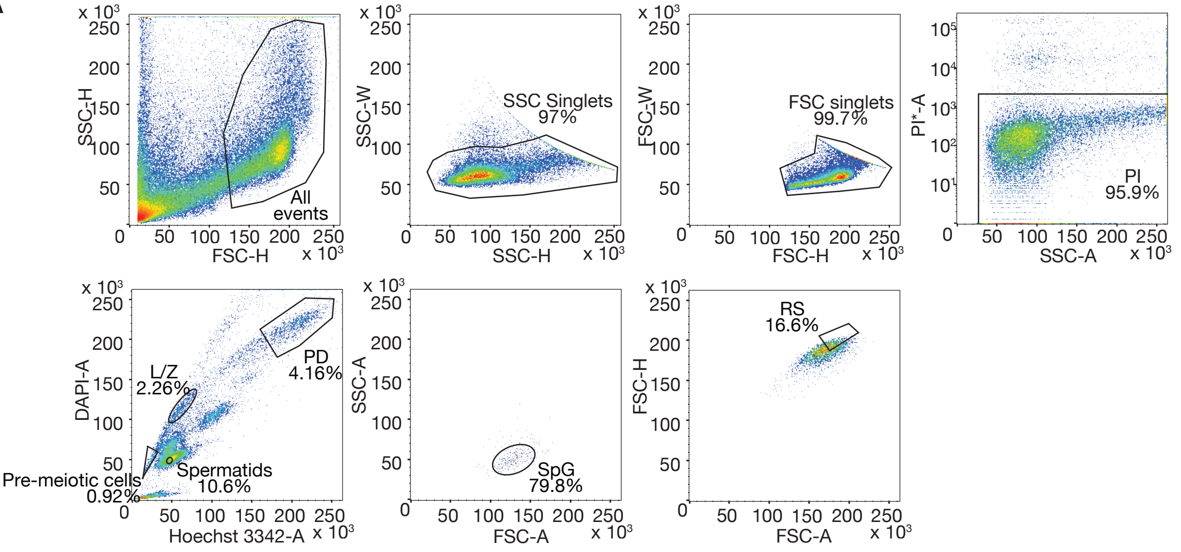

B

SpG gate; GCNA & DMRT1 staining

Animal #	GCNA$^+$/DMRT1$^+$	GCNA$^-$/DMRT1$^-$
1	41	1
2	56	5
3	64	2
4	61	1
5	70	8
6	46	1
%	95.3 ± 3.4	4.6 ± 3.4

Number of cells

L/Z gate; SYCP3 & γH2A.X staining

Animal #	SpG (SYCP3$^-$/γH2A.X$^-$)	L (SYCP3$^+$/γH2A.X$^+$)	Z (SYCP3$^+$/γH2A.X$^+$)	eP (SYCP3$^+$/γH2A.X$^+$)	P (SYCP3$^+$/γH2A.X$^+$)	D (SYCP3$^+$/γH2A.X$^+$)	RS (SYCP3$^-$/γH2A.X$^-$)	Sperm (SYCP3$^-$/γH2A.X$^-$)
1	4	28	29	7	0	0	0	4
2	10	50	27	11	0	0	0	3
3	3	47	26	8	0	0	0	7
4	11	90	62	14	2	0	0	5
5	4	46	18	14	0	0	1	10
6	2	48	23	6	0	0	1	2
%	5.2 ± 2.4	49.5 ± 5.8	29.4 ± 6.4	9.9 ± 2.6	0.2 ± 0.4	0 ± 0	0.4 ± 0.5	5.4 ± 3

Number of cells

P/D gate; SYCP3 & γH2A.X staining

Animal #	SpG (SYCP3$^-$/γH2A.X$^-$)	L (SYCP3$^+$/γH2A.X$^+$)	Z (SYCP3$^+$/γH2A.X$^+$)	eP (SYCP3$^+$/γH2A.X$^+$)	P (SYCP3$^+$/γH2A.X$^+$)	D (SYCP3$^+$/γH2A.X$^+$)	RS (SYCP3$^-$/γH2A.X$^-$)	Sperm (SYCP3$^-$/γH2A.X$^-$)
1	1	0	0	0	27	21	0	0
2	0	13	0	0	41	32	1	0
3	0	0	0	0	30	35	0	0
4	0	0	0	0	47	35	21	0
5	3	5	0	0	61	46	12	4
6	0	3	1	2	51	50	8	0
%	0.7 ± 1	3.6 ± 5.3	0.1 ± 0.3	0.3 ± 0.6	47.5 ± 3.5	41 ± 6.8	6.3 ± 7.2	0.5 ± 1.1

Number of cells

RS gate; SYCP3 & γH2A.X staining

Animal #	SpG (SYCP3$^-$/γH2A.X$^-$)	L (SYCP3$^+$/γH2A.X$^+$)	Z (SYCP3$^+$/γH2A.X$^+$)	eP (SYCP3$^+$/γH2A.X$^+$)	P (SYCP3$^+$/γH2A.X$^+$)	D (SYCP3$^+$/γH2A.X$^+$)	RS (SYCP3$^-$/γH2A.X$^-$)	Sperm (SYCP3$^-$/γH2A.X$^-$)
1	0	0	0	0	0	0	80	0
2	0	0	0	0	0	0	35	14
3	0	0	0	0	0	0	71	13
4	0	0	0	0	0	0	48	14
5	0	0	0	0	0	0	83	3
6	0	0	0	0	0	0	95	0
%	0	0	0	0	0	0	88.3 ± 11.2	11.6 ± 11.3

Number of cells

C

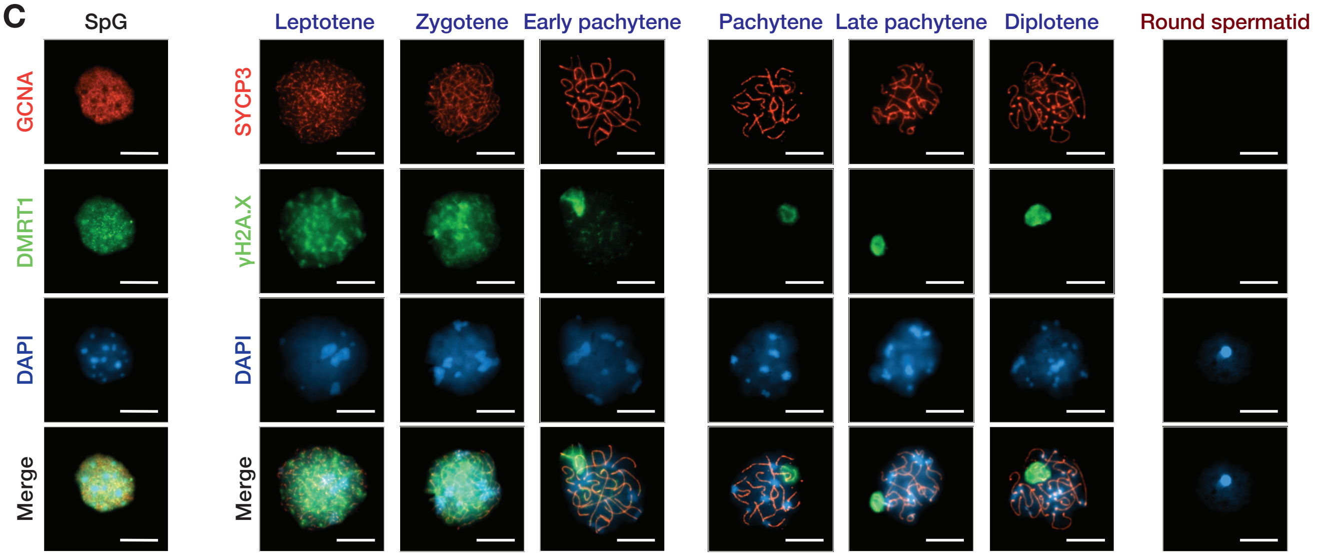

Appendix Figure S2. Strategy for purifying male germ cells using fluorescence-activated cell sorter (FACS). Related to Figures 1 and 2.

(A) Gating strategy used to purify spermatogonia (SpG), leptotene/zygotene spermatocytes (L/Z), pachytene/diplotene spermatocytes (P/D) and round spermatids (RS). Cells were stained with Hoechst 3342 and propidium iodide (PI). 488-nm laser was used to excite PI, while 405-nm laser was used to excite Hoechst 33342. The emission for PI was recorded using 700/54 nm bandpass filter, while the emission of Hoechst 33342 was recorded by 660/10-nm (red) and 448/45-nm (blue) bandpass filters.

(B) Datasets summarize counted cells per sorted gate. Six independent replicates were assessed per gate and the average percentage of each cell type calculated with standard deviation.

(C) Immunofluorescence staining for various germ cell markers from the chromosome spreads of four FACS-purified populations (SPG, L/Z, P/D, RS) to assess the purify of each gate. Anti-GCNA and anti-DMRT1 to assess SpG; anti-SYCP3, anti-γH2A.X to assess L/Z, P/D and RS gates. Cell pictures are ordered from left to right according to the trajectory of male germ cell development. Scale bars: 10 μm.

Säflund et al., Appendix Figure S3

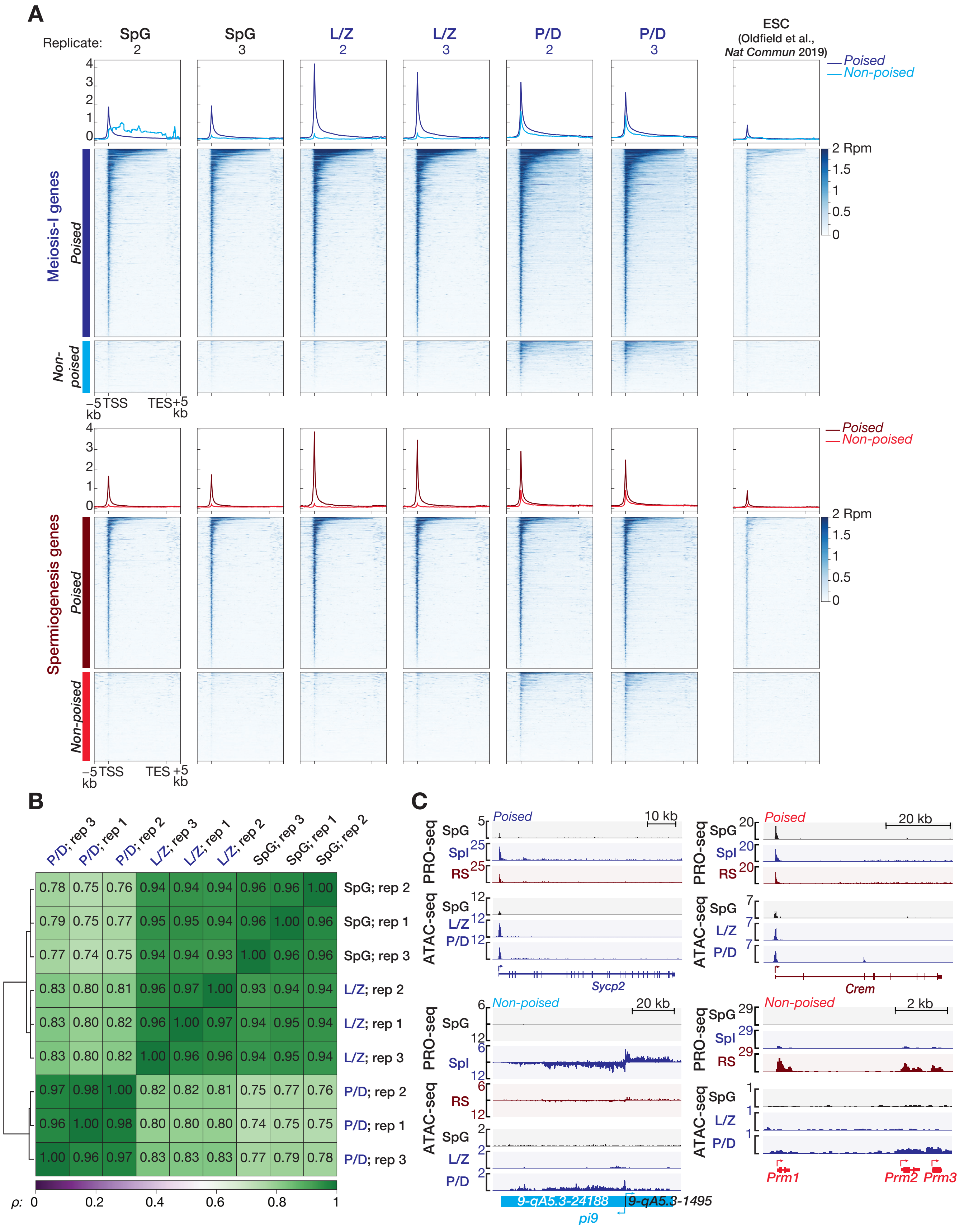

Appendix Figure S3. Poised genes have accessible chromatin around their promoter regions in spermatogonia. Related to Figures 1 and 2.

(A) Metagene plots (top) and heatmaps (bottom) show reads per million (Rpm)-normalized Assay for Transposase-Accessible Chromatin sequencing (ATAC-seq) signals in the –5 kb to +5 kb window flanking transcription start sites (TSSs) and transcription end sites (TESs) of poised and non-poised genes from the second and third replicates of spermatogonia (SpG), leptotene/zygotene (L/Z), and pachytene/diplotene (P/D). Embryonic stem cells (ESC (Oldfield *et al.*, 2019)) served as control.

(B) Heatmap shows spearman's correlation between the biological replicates of ATAC-seq from each cell population. Agreement between replicates; SpG Spearman's $\rho > 0.95$, L/Z Spearman's $\rho > 0.95$, P/D Spearman's $\rho > 0.96$).

(C) IGV views for PRO-seq (top) and ATAC-seq (bottom) signals at gene boundaries of exemplified poised and non-poised meiosis-I genes and spermiogenesis genes.

Poised meiosis I genes

	SpG rep 1			SpG rep 2			SpG rep 3		
Rank	Motif	Name	*p* value	Motif	Name	*p* value	Motif	Name	*p* value
1	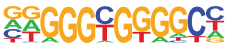	KLF1	10^{-384}	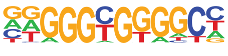	KLF1	10^{-360}	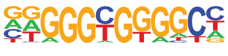	KLF1	10^{-380}
2	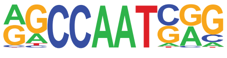	NFY	10^{-296}	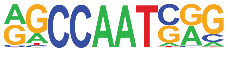	NFY	10^{-282}	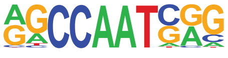	NFY	10^{-266}
3	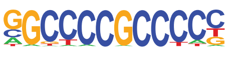	SP1	10^{-242}	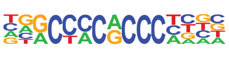	KLF3	10^{-216}	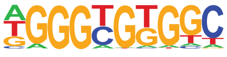	KLF5	10^{-258}
4	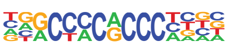	KLF3	10^{-234}	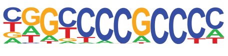	SP2	10^{-216}	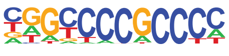	SP2	10^{-229}
5	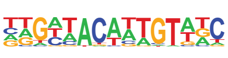	DMRT1	10^{-232}	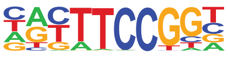	ELK4	10^{-209}	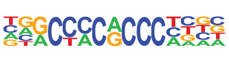	ELK3	10^{-213}

Poised spermiogenesis genes

	SpG rep 1			SpG rep 2			SpG rep 3		
Rank	Motif	Name	*p* value	Motif	Name	*p* value	Motif	Name	*p* value
1	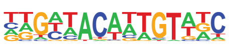	DMRT6	10^{-217}	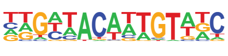	DMRT6	10^{-171}	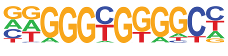	KLF1	10^{-198}
2	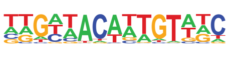	DMRT1	10^{-205}	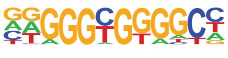	KLF1	10^{-169}	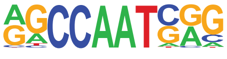	NFY	10^{-181}
3	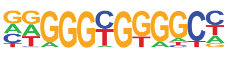	KLF1	10^{-187}	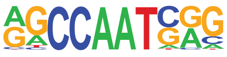	NFY	10^{-162}	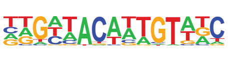	DMRT6	10^{-156}
4	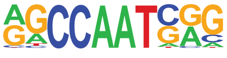	NFY	10^{-169}	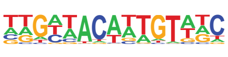	DMRT1	10^{-156}	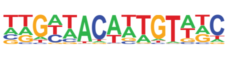	DMRT1	10^{-156}
5	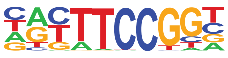	ELK4	10^{-128}	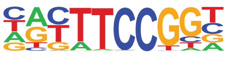	ELK4	10^{-126}	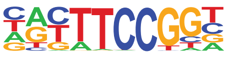	ELK4	10^{-135}

Appendix Figure S4. HOMER motif analysis around the promoters of poised genes. Related to Figure 2.

Datasets summarize the top-five scoring motifs obtained by HOMER motif analysis under ATAC-seq peaks within ± 2 kb of poised gene promoters. Top, poised meiosis-I genes. Bottom, poised spermiogenesis genes.

Säflund et al., Appendix Figure S5

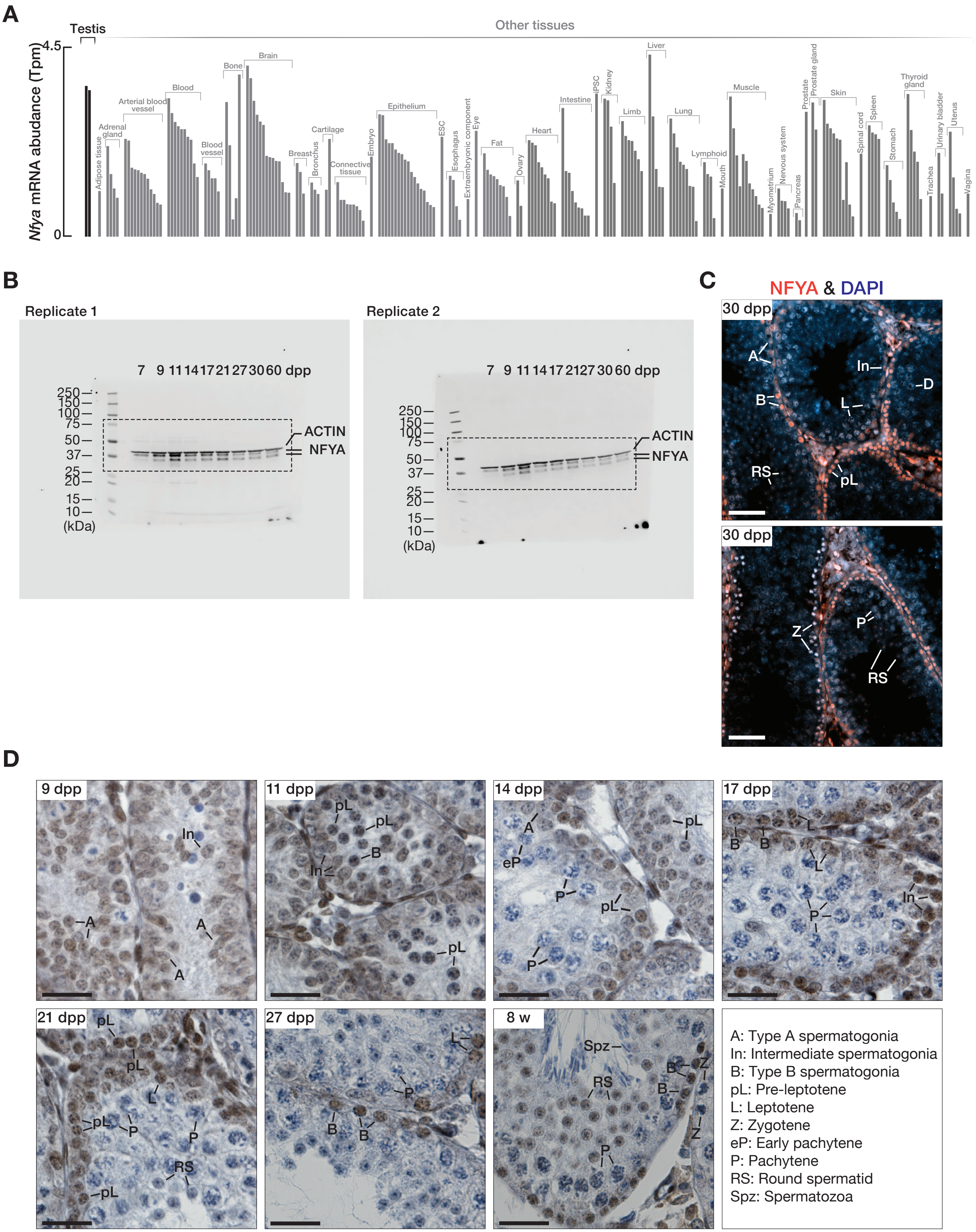

Appendix Figure S5. Temporal expression of NFYA in the testes of wild-type mouse. Related to Figure 2.

(A) Bars show transcripts per million (Tpm)-normalized mRNA abundance of *Nfya* across different mouse tissues. Data is from mouse Encyclopedia of DNA Elements (ENCODE).

(B) Western blot shows NFYA protein abundance in the testes from stage mice (i.e., 7, 9, 11, 14, 17, 21, 27, 30 and 60 days postpartum [dpp]). ACTIN used as loading control. Each lane contained 75 μg total testis protein isolate.

(C) Immunofluorescence staining against NFYA on the testis section from 30 dpp mouse. A, type A spermatogonia; In, intermediate spermatogonia; B, type B spermatogonia; pL, preleptotene; L, leptotene; Z, zygotene; P, pachytene; D, Diplotene; RS, round spermatid. Scale bars: 50 μm.

(D) Immunohistochemical (IHC) detection of NFYA on Bouin fixed testes sections from stage mice. A, type A spermatogonia; In, intermediate spermatogonia; B, type B spermatogonia; pL, preleptotene; L, leptotene; Z, zygotene; eP, early pachytene; P, pachytene; RS, round spermatid; Spz, Spermatozoa. Scale bars: 25 μm.

Säflund et al., Appendix Figure S6

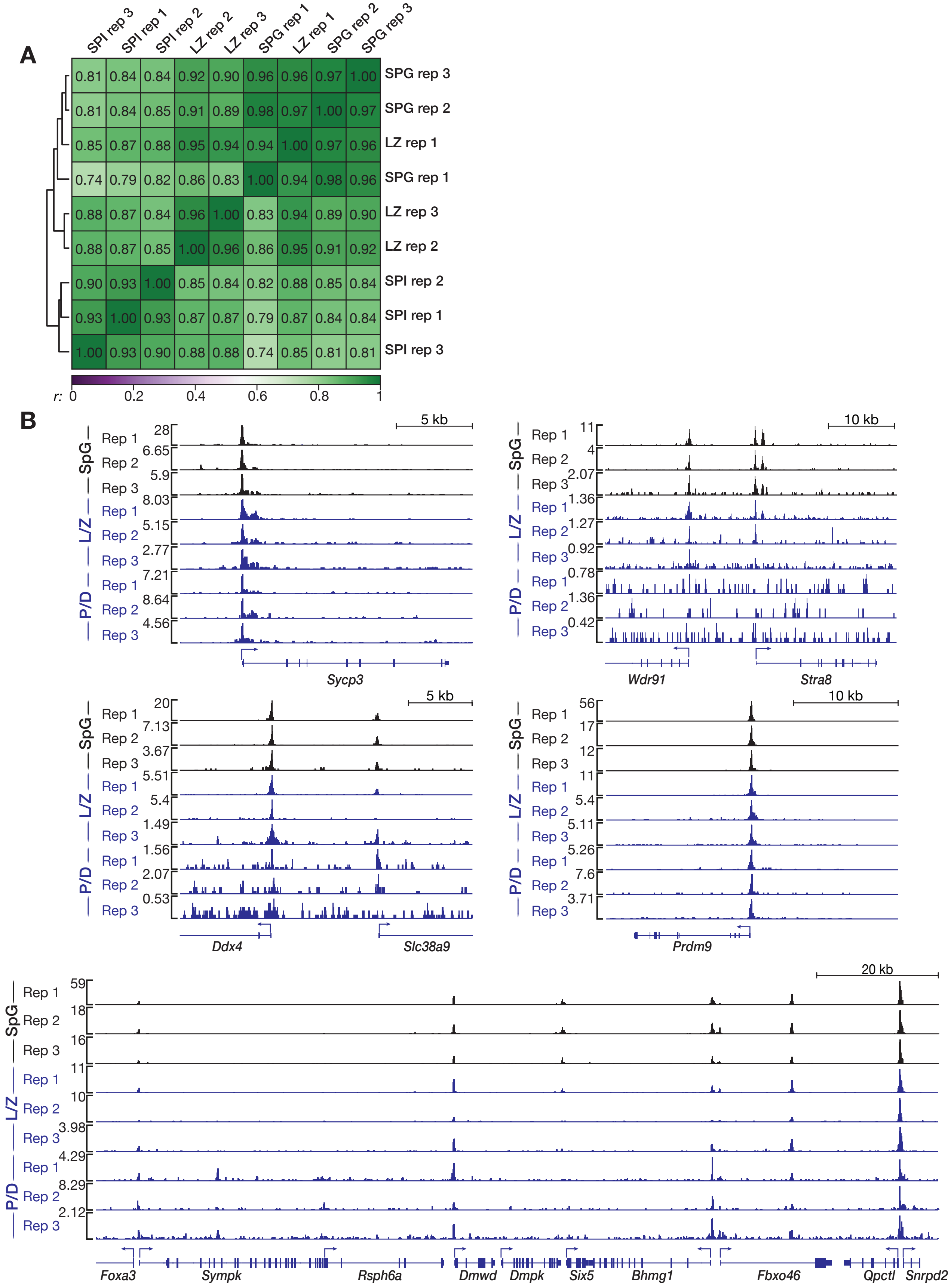

Appendix Figure S6. Correlation between NFYA CUT&RUN replicates. Related to Figure 2.

(A) Heatmap shows Pearson correlation coefficient (*r*) between the biological replicates of NFYA CUT&RUN from each cell population. Agreement between replicates; SpG Pearson correlation coefficient (*r*) > 0.95, L/Z Pearson correlation coefficient (*r*) > 0.93, P/D Pearson correlation coefficient (*r*) > 0.89).

(B) IGV views for NFYA CUT&RUN signals form all biological replicates at gene boundaries of exemplified genes.

Säflund et al., Appendix Figure S7

A

NFYA CUT&RUN from SpG cells

Sample	# of NFYA peaks	% of peaks near TSSs	# of protein-coding genes	# of non-coding genes	% of peaks at enhancers (marked by H3K4me1)
Replicate 1	61,293	42.2%	10,603	2,999	3.6%
Replicate 2	5,213	88.1%	3,744	366	1.6%
Replicate 3	4,077	80.0%	3,006	257	1.4%

NFYA CUT&RUN from L/Z cells

Sample	# of NFYA peaks	% of peaks near TSSs	# of protein-coding genes	# of non-coding genes	% of peaks at enhancers (marked by H3K27Ac)
Replicate 1	3,560	90.6%	2,758	225	13.9%
Replicate 2	1,696	92.9%	1,366	105	16.3%
Replicate 3	933	95.5%	795	51	13.5%

NFYA CUT&RUN from P/D cells

Sample	# of NFYA peaks	% of peaks near TSSs	# of protein-coding genes	# of non-coding genes	% of peaks at enhancers (marked by H3K27Ac)
Replicate 1	9,896	40.4%	2,814	562	4.6%
Replicate 2	4,354	58.6%	1,953	273	7.3%
Replicate 3	13,853	26.1%	2,139	743	2.8%

B

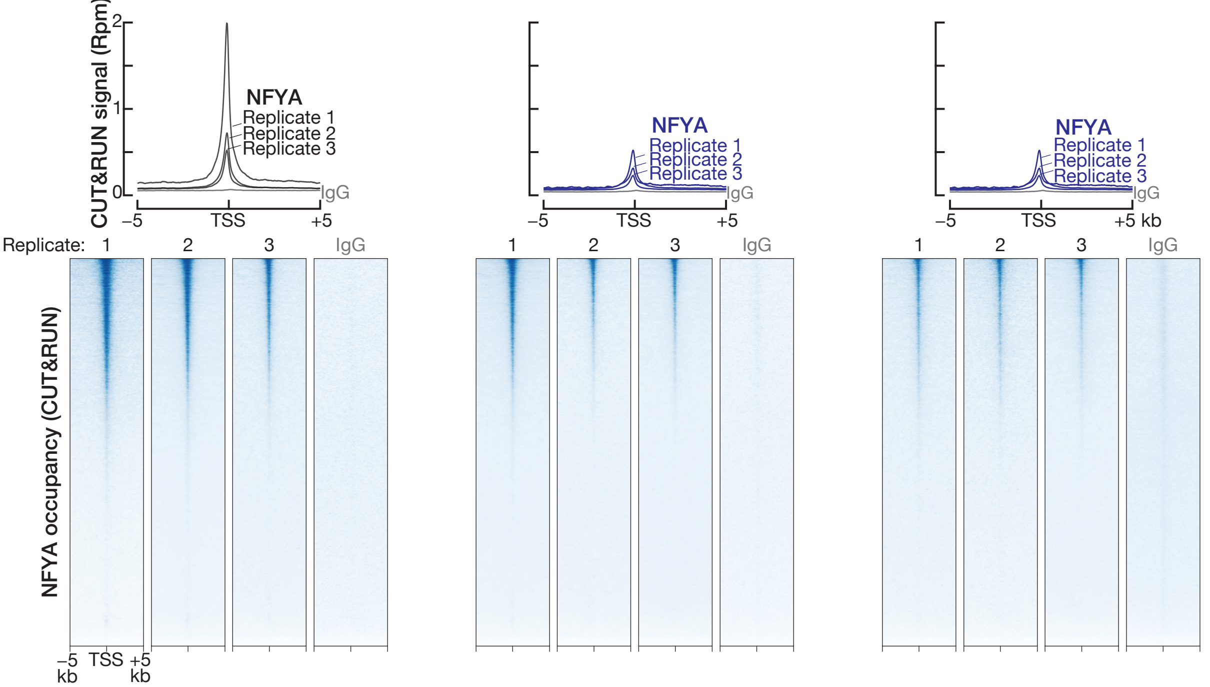

C

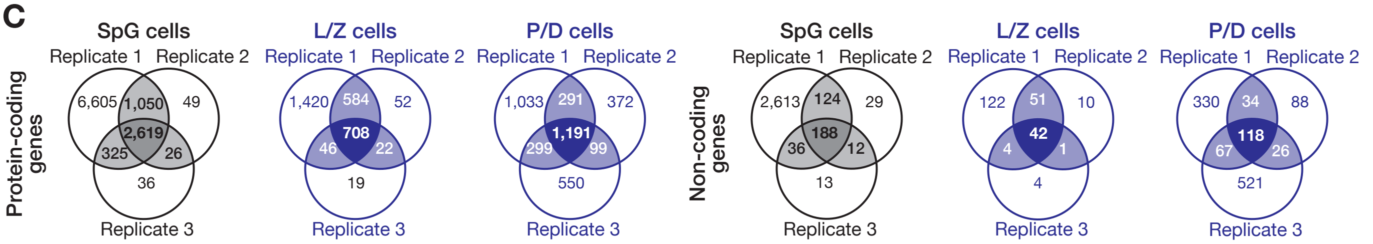

Appendix Figure S7. NFYA binds the promoters of poised genes in spermatogonia. Related to Figure 2.

(A) Datasets summarize genome-wide significant NFYA CUT&RUN peaks in the three replicates of spermatogonia (SpG), leptotene/zygotene spermatocytes (L/Z), and pachytene/diplotene spermatocytes (P/D).

(B) Metaplots (top) and heatmaps (bottom) show average reads per million (Rpm)-normalized NFYA CUT&RUN signals in the –5 kb to +5 kb window flanking transcription start sites (TSSs) of all mouse RefSeq genes in SpG, L/Z, and P/D. IgG used for negative control.

(C) Venn diagrams show number of overlapping protein- and non-coding genes across three biological replicates.

Säflund et al., Appendix Figure S8

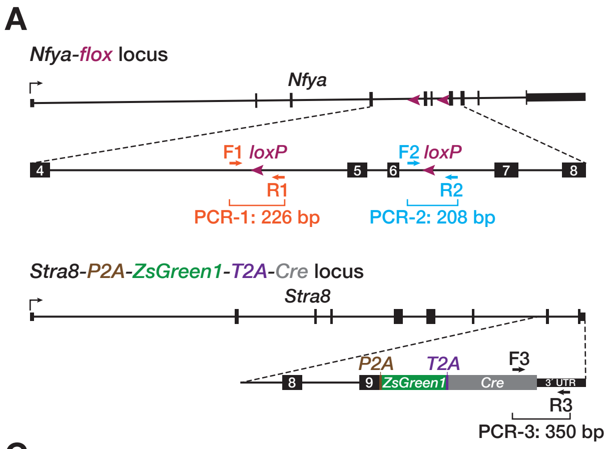

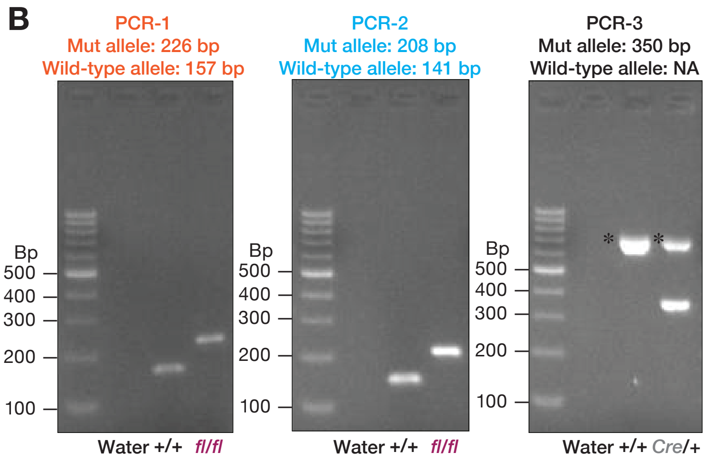

C

Breeders #1	♂ Nfya$^{fl/+}$; Stra8-P2A-ZsGreen1-T2A-Cre$^{Cre/+}$ × ♀ Nfya$^{fl/+}$ and ♀ Nfya$^{fl/+}$					
122 total mice	fl/fl; +/+	fl/fl; Cre/+	fl/+; +/+	fl/+; Cre/+	+/+; +/+	+/+; Cre/+
Number of mice	0	8	19	14	69	12
Expected ratio	0.125	0.125	0.25	0.25	0.125	0.125
Observed ratio	0	0.07	0.16	0.11	0.6	0.1

Breeders #2	♂ Nfya$^{fl/+}$; Stra8-P2A-ZsGreen1-T2A-Cre$^{Cre/+}$ × ♀ Nfya$^{fl/+}$ and ♀ Nfya$^{fl/fl}$					
38 total mice	fl/fl; +/+	fl/fl; Cre/+	fl/+; +/+	fl/+; Cre/+	+/+; +/+	+/+; Cre/+
Number of mice	9	3	8	12	1	5
Expected ratio	0.125/ 0.25	0.125/ 0.25	0.25	0.25	0.125/ 0	0.125/ 0
Observed ratio	0.24	0.08	0.21	0.32	0.03	0.13

Breeders #3	♂ Nfya$^{fl/+}$; Stra8-P2A-ZsGreen1-T2A-Cre$^{Cre/+}$ × ♀ Nfya$^{fl/fl}$ and ♀ Nfya$^{fl/fl}$					
22 total mice	fl/fl; +/+	fl/fl; Cre/+	fl/+; +/+	fl/+; Cre/+	+/+; +/+	+/+; Cre/+
Number of mice	2	6	7	7	0	0
Expected ratio	0.25	0.25	0.25	0.25	0	0
Observed ratio	0.09	0.27	0.32	0.32	0	0

Breeders #4	♂ Nfya$^{fl/fl}$ × ♀ Nfya$^{fl/+}$; Stra8-P2A-ZsGreen1-T2A-Cre$^{Cre/+}$ and ♀ Nfya$^{fl/+}$; Stra8-P2A-ZsGreen1-T2A-Cre$^{Cre/+}$					
22 total mice	fl/fl; +/+	fl/fl; Cre/+	fl/+; +/+	fl/+; Cre/+	+/+; +/+	+/+; Cre/+
Number of mice	9	5	5	3	0	0
Expected ratio	0.25	0.25	0.25	0.25	0	0
Observed ratio	0	0.07	0.16	0.11	0.6	0.1

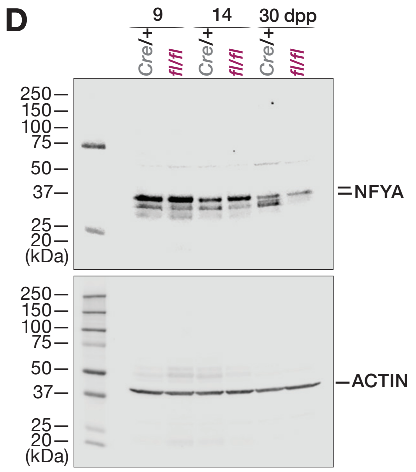

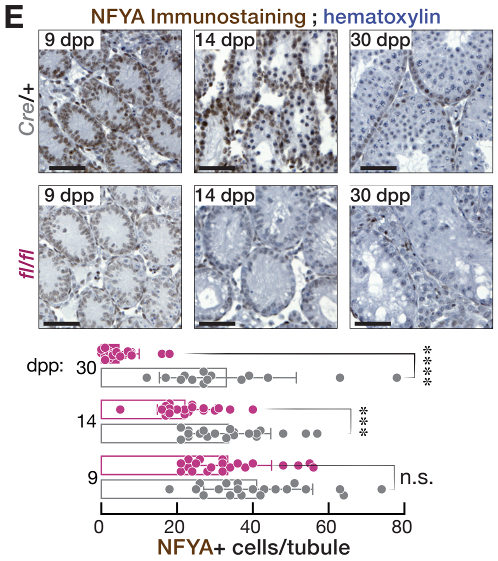

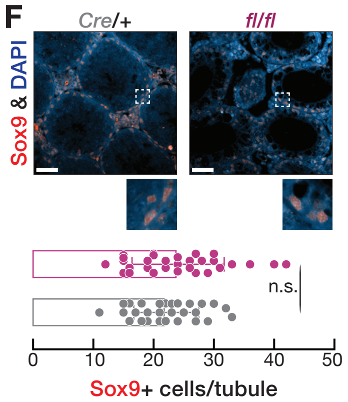

Appendix Figure S8. Male germ cell-specific deletion of *Nfya*. Related to Figure 3.

(A) Strategy to delete *Nfya* in male germ cells using Stra8-Cre-loxP system.

(B) Agarose gel electrophoresis images for genotyping of mutants by PCR. Each *loxp* site flanking the exon was validated by PCR amplification (PCR-1 and PCR-2 primers are highlighted in red and light blue) in mice with *Stra8-Cre* transgene which was also confirmed by PCR (PCR-3 primers are highlighted in black). * Represents internal control PCR bands.

(C) Datasets show number and frequency of pups with all possible genotypes from four different breading pairs. Highlighted are the number of pups with homozygous deletion.

(D) Western blot images show NFYA abundance in *Cre/+* and *fl/fl* mice at 9, 14 and 30 days postpartum (dpp). ACTIN served as loading control. Each lane contained 75 µg testis protein.

(E) Immunohistochemical (IHC) detection of NFYA on Bouin fixed testes sections from from *Cre/+* and *fl/fl* mice at 9, 14, 30 dpp. Scale bars: 50 µm. Bars represent quantification for NFYA positive cells per tubule from two or three independent biological replicates for each staining (n = 3). Whiskers show standard deviation and center of the whiskers represents the mean. Significance is measured using two-sided unpaired student t-test. 14 dpp, *fl/fl* vs *Cre/+* mice p = 0.0004; 30 dpp, *fl/fl* vs *Cre/+* mice $p = 1.454 \times 10^{-7}$

(F) Immunofluorescence (IF) detection of SOX9 on Bouin fixed testes sections from from *Cre/+* and *fl/fl* mice 30 dpp (n = 3). Bars represent quantification for positive cells per tubule from three independent biological replicates. Whiskers represent standard deviation and center of the whiskers represents the mean. Significance is measured using two-sided unpaired student t-test. Scale bars: 50 µm.

Säflund et al., Appendix Figure S9

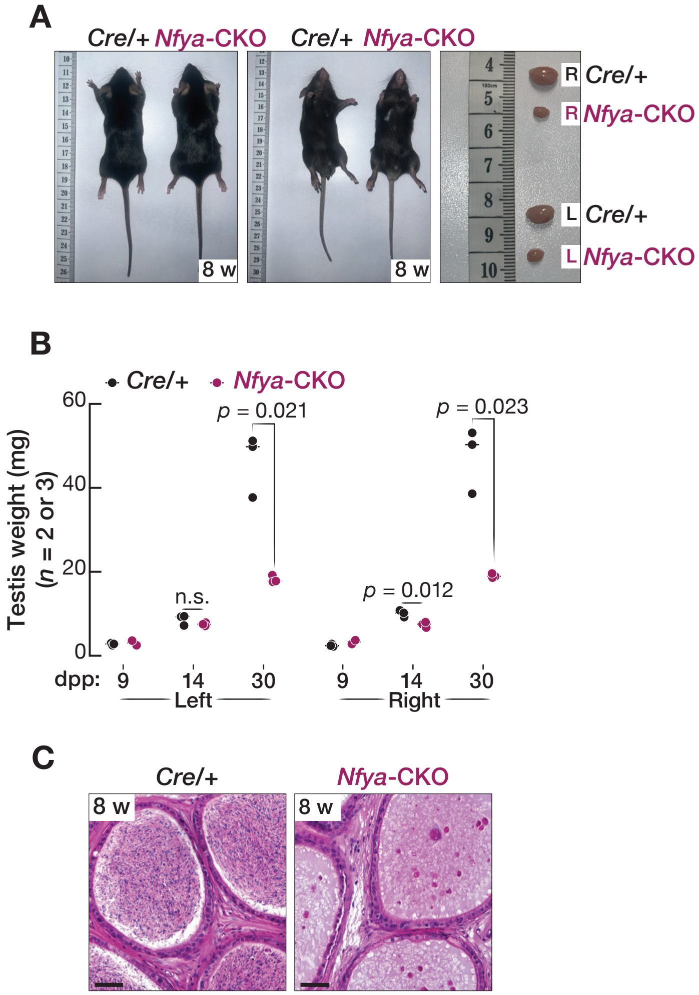

Appendix Figure S9. NFYA deficient male germ cells have reduced testis size and no sperm in epididymis. Related to Figure 3.

(A) Comparison of testes from 8-week-old *Cre/+* and *Nfya-CKO* mice.

(B) Relative testes weights in *Cre/+* and *Nfya-CKO* mice at 9 days postpartum (dpp), 14 dpp, and 30 dpp. (n = 2 or n = 3). Significance is measured using two-sided paired student t-test.

(C) Hematoxylin and eosin staining of the caudal epididymis sections from 8-week-old *Cre/+* and *Nfya-CKO* mice. Scale bars: 50 μm.

Säflund et al., Appendix Figure S10

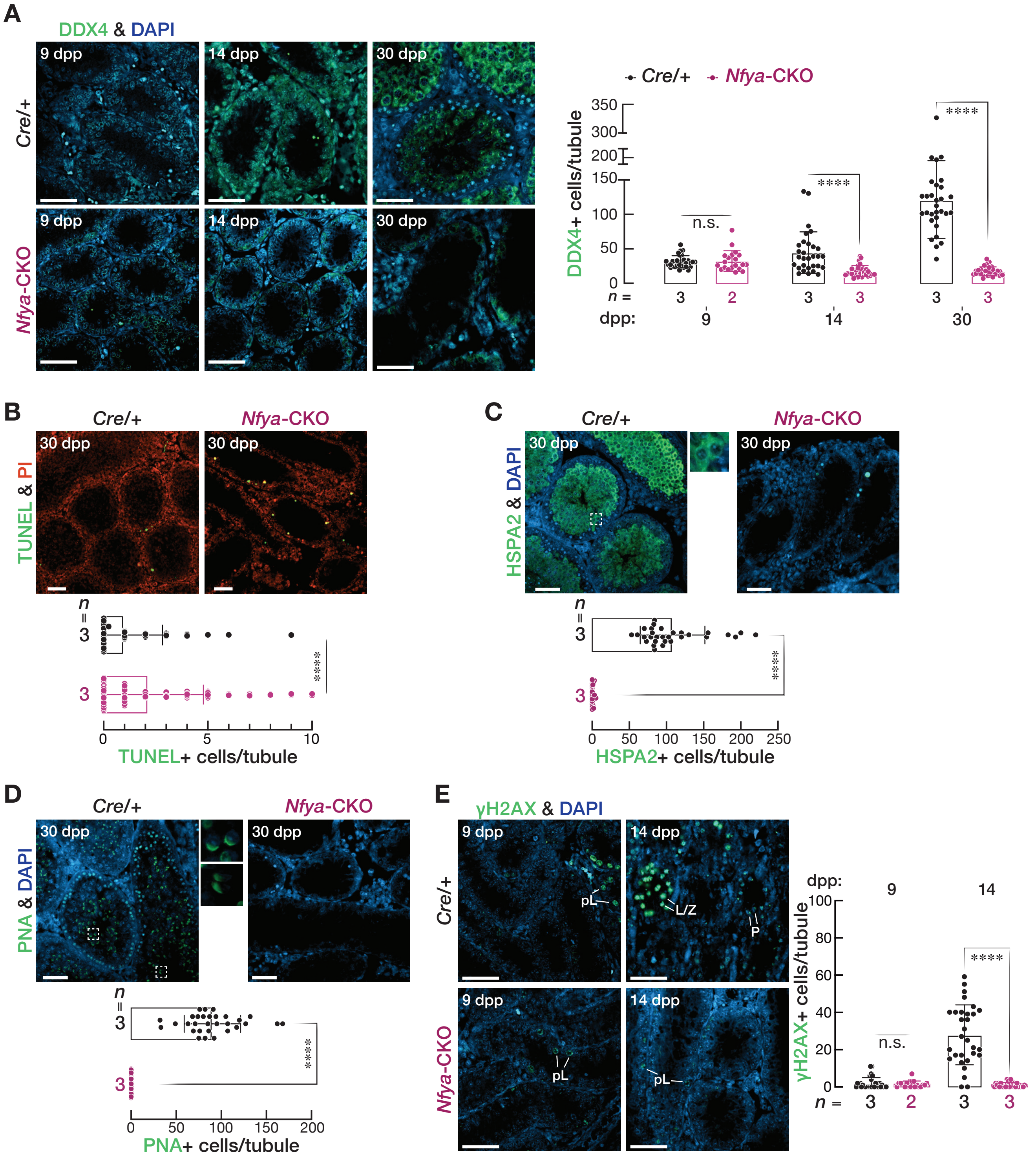

Appendix Figure S10. NFYA deficient male germ cells arrest at meiotic entry. Related to Figure 3.

(A-E) Immunofluorescence staining of testes sections from 9, 14 and 30 dpp *Cre/+* and *Nfya*-CKO for **(A)** DDX4 (9 dpp, *Cre/+*, $n = 3$; 9 dpp, *Nfya*-CKO $n = 2$, $p = 0.7254$; 14dpp, $n = 3$, $p = 1.74 \times 10^{-5}$; 30dpp, $n = 3$, $p = 2.07 \times 10^{-14}$), **(B)** TdT-mediated dUTP Nick-End Labeling (TUNEL) ($n = 3$, $p = 1.34 \times 10^{-5}$), **(C)** HSPA2 ($n = 3$, $p = 1.27 \times 10^{-19}$)**, (D)** PNA ($n = 3$, $p = 1.12 \times 10^{-22}$) and **(E)** γH2A.X (9 dpp, *Cre/+*, $n = 3$; 9 dpp, *Nfya*-CKO $n = 2$, $p = 0.5957$; 14 dpp, $n = 3$ $p = 8.84 \times 10^{-13}$). Bars represent quantification for positive cells per tubule from three independent biological replicates. Whiskers represent standard deviation and center of the whiskers represents the mean. Significance is measured using two-sided unpaired student t-test. Scale bars: 50 µm.

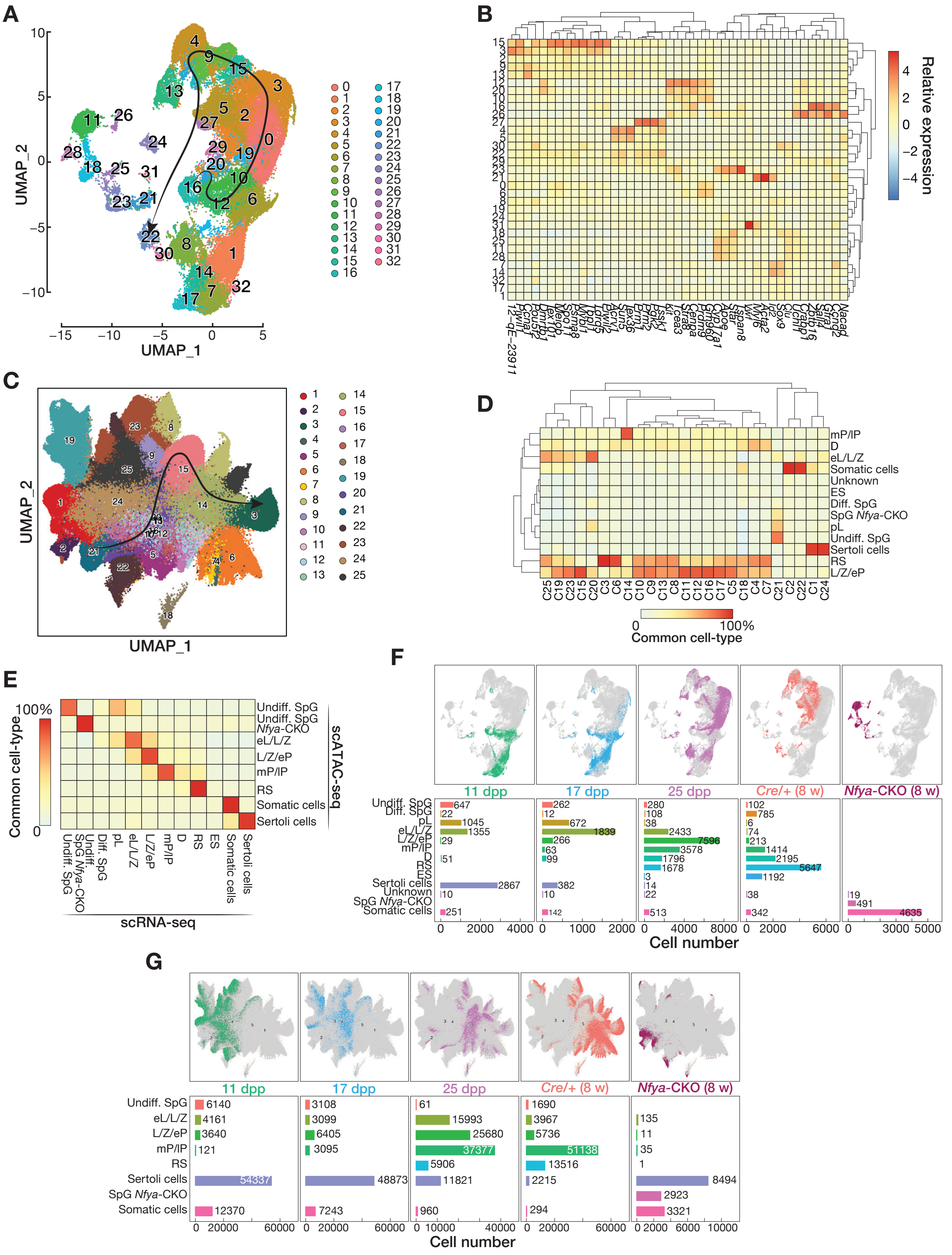
A
UMAP_1
UMAP_2
B
Relative expression
C
UMAP_1
UMAP_2
D
mP/lP
D
eL/L/Z
Somatic cells
Unknown
ES
Diff. SpG
SpG Nfya-CKO
pL
Undiff. SpG
Sertoli cells
RS
L/Z/eP
Common cell-type
E
Common cell-type
scATAC-seq
scRNA-seq
F
11 dpp
17 dpp
25 dpp
Cre/+ (8 w)
Nfya-CKO (8 w)
Cell number
G
11 dpp
17 dpp
25 dpp
Cre/+ (8 w)
Nfya-CKO (8 w)
Undiff. SpG
eL/L/Z
L/Z/eP
mP/lP
RS
Sertoli cells
SpG Nfya-CKO
Somatic cells
Cell number

Appendix figure S11. Identification of cell populations of the testes from 8-week-old Cre/+ and Nfya-CKO mice along with wild-type staged mice using simultaneous single-cell (sc) RNA-seq and ATAC-seq. Related to Figures 4, 5, and 6.

(A) Uniform manifold approximation and projection (UMAP) space visualizes 32 clusters obtained by dimension reduction analysis on 45,250 individual cells from 11, 17, 25 dpp, *Cre/+* and *Nfya-CKO* mice. Developmental trajectory for germ cells is shown.

(B) Unsupervised hierarchical clustering of 32 clusters based on the relative expression of cell markers.

(C) UMAP space visualizes 25 cell clusters defined by the chromatin accessibility obtained from scATAC-seq of 343,866 individual cells from 11, 17, 25 dpp, *Cre/+* and *Nfya-CKO* mice. Developmental trajectory for germ cells is shown.

(D) Heatmap shows the percentage of cell, whose type is defined by scRNA-seq, in clusters obtained by scATAC-seq.

(E) Heatmap shows the percentage of cells that are classified in the same cell-type in both scRNA-seq and scATAC-seq.

(F) UMAP spaces (top) illustrate the identity of cellular diversity in each mouse compared to the background of remaining mice (grey color). Bars (bottom) represent the total number of individual cells belonging to each major cell population defined in panel A in each mouse.

(G) UMAP spaces (top) illustrate identity of cellular diversity obtained from scATAC-seq in each mouse compared to the background of remaining mice (grey color). Bars (bottom) represent the total number of individual cells belonging to each major cell population.
